# Integrated electronic/fluidic microneedle system for glucose sensing and insulin delivery

**DOI:** 10.7150/thno.92910

**Published:** 2024-02-11

**Authors:** Xinshuo Huang, Baoming Liang, Shuang Huang, Zhengjie Liu, Chuanjie Yao, Jingbo Yang, Shantao Zheng, Feifei Wu, Wan Yue, Ji Wang, Huijiuan Chen, Xi Xie

**Affiliations:** 1 State Key Laboratory of Optoelectronic Materials and Technologies; Guangdong Province Key Laboratory of Display Material and Technology; School of Electronics and Information Technology; Sun Yat-Sen University, Guangzhou, 510006, China.; 2 Guangdong Provincial Key Laboratory of Sensor Technology and Biomedical Instrument, School of Biomedical Engineering, Sun Yat-Sen University, Shenzhen, 518107, China.; 3 School of Biomedical Engineering, Shenzhen Campus of Sun Yat-Sen University, Shenzhen, 518107, China.; 4 Pazhou Lab, Guangzhou, 510330, China.; 5 School of Materials Science and Engineering, Sun Yat-Sen University, Guangzhou, 510006, China.; 6 The First Affiliated Hospital of Sun Yat-Sen University, Sun Yat-Sen University, Guangzhou, 510006, China.

**Keywords:** wired enzyme, electronic/fluidic microneedle, glucose sensing, insulin delivery, wearable system

## Abstract

**Background:** Precise and dynamic blood glucose regulation is paramount for both diagnosing and managing diabetes. Continuous glucose monitoring (CGM) coupled with insulin pumps forms an artificial pancreas, enabling closed-loop control of blood glucose levels. Indeed, this integration necessitates advanced micro-nano fabrication techniques to miniaturize and combine sensing and delivery modules on a single electrode. While microneedle technology can mitigate discomfort, concerns remain regarding infection risk and potential sensitivity limitations due to their short needle length.

**Methods:** This study presents the development of an integrated electronic/fluidic microneedle patch (IEFMN) designed for both glucose sensing and insulin delivery. The use of minimally invasive microneedles mitigates nerve contact and reduces infection risks. The incorporation of wired enzymes addresses the issue of "oxygen deprivation" during glucose detection by decreasing the reliance on oxygen. The glucose-sensing electrodes employ wired enzyme functionalization to achieve lower operating voltages and enhanced resilience to sensor interference. The hollow microneedles' inner channel facilitates precise drug delivery for blood glucose regulation.

**Results:** Our IEFMN-based system demonstrated high sensitivity, selectivity, and a wide response range in glucose detection at relatively low voltages. This effectively reduced interference from both external and internal active substances. The microneedle array ensured painless and minimally invasive skin penetration, while wired enzyme functionalization not only lowered sensing potential but also improved glucose detection accuracy. In vivo, experiments conducted in rats showed that the device could track subcutaneous glucose fluctuations in real-time and deliver insulin to regulate blood glucose levels.

**Conclusions:** Our work suggests that the IEFMN-based system, developed for glucose sensing and insulin delivery, exhibits good performance during in vivo glucose detection and drug delivery. It holds the potential to contribute to real-time, intelligent, and controllable diabetes management.

## Introduction

Diabetes, characterized by elevated blood glucose levels resulting from defective insulin secretion or impaired biological function 1-3, adversely affects normal tissue function by limiting cellular access to glucose, the primary energy source for daily metabolic processes 4-6. It has been established that prolonged hyperglycemia poses a significant risk, leading to chronic damage or dysfunction in vital tissues such as eyes, kidneys, heart, blood vessels, and nerves, thereby posing a threat to individuals' lives 7-9. Individuals with diabetes mellitus utilize self-monitoring of blood glucose (SMBG) technologies, primarily employing finger prick sampling, to obtain real-time glycemic data. Subsequently, manual insulin injections are administered for optimal glucose regulation 10-13. However, the need for frequent SMBG operations can cause tissue trauma and discomfort 14, 15 , and the intermittent nature of blood sample collection fails to accurately reflect the body's glucose fluctuations 16, 17. Traditional insulin injection methods with metal needles also carry the potential risk of tissue trauma and infection 18-20. Efforts to develop non-invasive blood glucose measuring technologies, including optical, metabolic thermal, and electrochemical methods using human saliva, tears, and sweat 21-24 , have faced challenges in gaining clinical acceptance due to difficulties in specific glucose measurement or the unclear correlation between glucose concentrations in blood and other body fluids 25-27. Continuous Glucose Monitoring (CGM) technology, relying on implantable electrodes, has shown promising progress in clinical trials and market applications 28-30. Glucose oxidase (GOx) is implanted onto these electrodes to catalyze the oxidation of glucose in interstitial fluid, converting glucose concentrations into electrical signals. This process involves monitoring changes in the current signal during the oxidation reaction, allowing for the extrapolation of glucose levels 7, 31, 32.

The development of CGM technology has undergone two iterations in the design of glucose-sensing electrodes 33, 34. In the first generation, glucose biosensors primarily relied on estimating glucose concentration through hydrogen peroxide (H_2_O_2_) production by GOx, utilizing dissolved oxygen. During this process, the FAD cofactor of GOx is reduced to FADH_2_, which then reacts with O_2_ and is oxidized back to FAD, generating H_2_O_2_. The use of H_2_O_2_ as a transmission mediator for electron transfer in the first-generation implantable glucose sensing electrodes led to reduced detection accuracy due to "oxygen deprivation" 3, 14. Additionally, these electrodes operated at high voltages (above 0.5 V) and were susceptible to interference from active substances like ascorbic acid, uric acid, amino acids, and lactic acid 35-37. In the context of second-generation glucose biosensors, a redox mediator is employed to facilitate the transfer of electrons from the active center of the enzyme to the electrode surface, effectively replacing the role of oxygen (O_2_) seen in first-generation biosensors. This redox mediator operates by transferring electrons from the active site to the electrode surface. Specifically, the second generation of implantable glucose-sensing electrodes utilizes small molecules or polymers with redox capabilities as electron mediators. Notably, the "wired enzyme" approach involves the use of redox polymers (e.g., [Poly(vinylpyridine)] or [Poly(vinylimidazole)]) as a structural backbone to connect the active site of GOx with the electrode surface, forming a three-dimensional network structure. This configuration serves to reduce the distance between the FAD active center of the enzyme and the redox center of the polymer. Sensors developed using the "wired enzyme" technology exhibit advantages such as higher outputs, shorter response times, and secure anchoring of the mediator to the electrode surface. This is achieved by connecting the GOx interspersed in the polymer network to the electrode surface through hydrophilic polymer long chains that are covalently linked with osmium complexes 38, 39. The redox reaction involving Os^3+^/Os^2+^ represents an effective mechanism for transferring electrons from the enzyme to the electrode surface. This process not only circumvents the participation of oxygen but also fundamentally addresses the issue of "oxygen deprivation" in the interstitial fluid. Moreover, the integration of an implantable continuous glucose monitor with an insulin pump allows for intelligent drug delivery, facilitating timely regulation of blood glucose levels in the patient's body. This integration forms a Closed-Loop System. However, it is important to note that the electrodes of implantable CGM devices and insulin pump needles typically have lengths exceeding 5 mm. This length may cause discomfort and pain to patients easily after penetrating the skin, posing a potential risk of bleeding and infection.

The past few years have witnessed a burgeoning interest in microneedles (MN) with needle tips ranging from 500 to 800 μm in length 40, 41. These microneedles can penetrate the stratum corneum without contacting subcutaneous nerves or causing damage to capillaries, effectively minimizing the risk of bleeding and pain 42, 43. As an efficient and minimally invasive technology, microneedle applications have expanded into biomarker detection and drug delivery 44, 45. For instance, Gu et al. devised a microneedle patch loaded with glucose-responsive vesicles and insulin drugs. This patch dissolves rapidly upon penetration, enabling insulin delivery into subcutaneous tissue and facilitating intelligent blood glucose regulation. In experiments with type I diabetic rats, this microneedle patch successfully maintained blood glucose levels for 21 days. Li et al. combined mesoporous microneedles with iontophoresis technology to achieve in situ sensing and drug delivery in diabetes 46. Yang et al. designed a microneedle therapy integrated system with a bionic structure, capable of painlessly penetrating the skin for subcutaneous glucose detection, monitoring physiological ions fluctuation, and performing insulin delivery 47. However, existing integrated microneedle-based detection-therapy devices typically assemble microneedle sensing modules and microneedle drug delivery modules separately. Fabricating multifunctional microneedle structures faces challenges, including the simultaneous implementation of sensing and drug delivery functions onto a tiny microneedle. This challenge is crucial for further miniaturization and achieving high integration of the device. Firstly, distinguishing the enzyme electrochemical sensing module and fluid delivery module on the same microneedle patch demands advanced micro-nano processing technology. Secondly, the multifunctionality of the microneedle, combined with the incorporation of a micro-channel module in the device, limits the sensing area of the electrode, resulting in reduced signal strength and sensitivity. Thirdly, the sensing signal of microneedle electrodes can be easily influenced by the concurrent delivery of drug molecules in the same microneedle patch.

**Figure 1:**
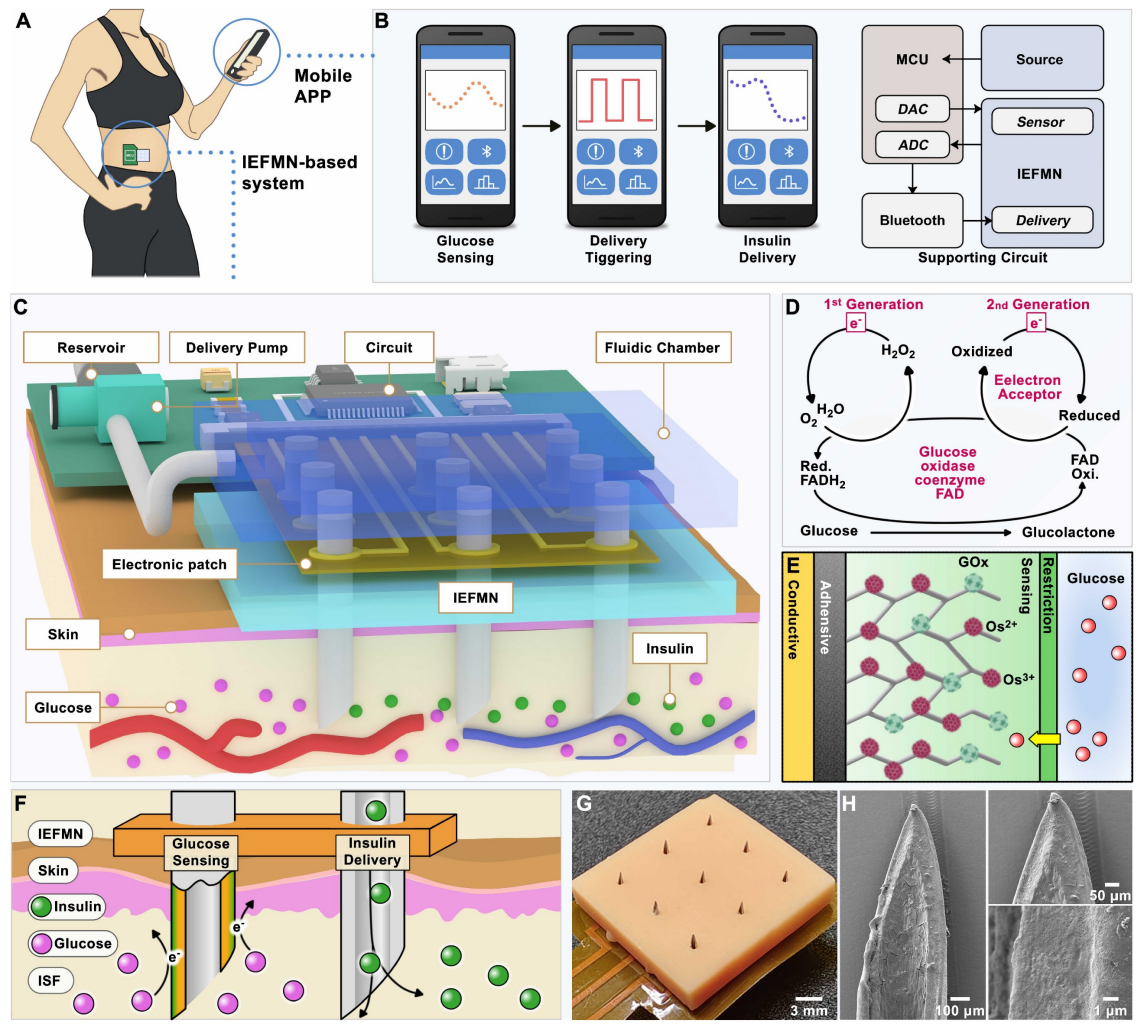
** Schematic diagram of the system based on integrated electronic/fluidic microneedle patch for glucose sensing and insulin delivery.** (A) Schematic illustration of the IEFMN-based system. (B) Smart phone application for displaying blood glucose level data and providing timely alerts. (C) Components of system based on integrated electronic/fluidic microneedle patch. (D) Sensing mechanism of both first and second-generation glucose sensing electrodes. (E) Schematic showing the multi-layer structure of the integrated electronic/fluidic microneedle electrode. (F) Illustrations depicting the function of IEFMN in transdermal glucose detection and drug delivery. (G) Optical photograph of IEFMN. (H) Morphological characterization of the sensing module within IEFMN.

Herein, we introduced an innovative integrated electronic/fluidic microneedle patch system designed for both glucose sensing and insulin delivery (IEFMN, **Figure 1**). The incorporation of a hollow microneedle electrode ensures a minimally invasive and safe transdermal detection method, thereby reducing the risk of pain or potential infection associated with prolonged electrode use. The IEFMN, equipped with a micro-channel module enabling simultaneous transdermal glucose sensing and drug delivery on the same microneedle patch, is developed through the sequential integration of individually fabricated and functionalized hollow microneedle electrodes. The application of wired enzyme functionalization on the microneedle sidewall facilitates rapid electron transfer from the active center of GOx to the electrode interior. This results in lower operating voltages for glucose sensing and effectively avoids interference from active substances. *In vitro* experiments demonstrated that the IEFMN could exhibit high accuracy, a broad linear range, and selectivity in glucose sensing while demonstrating the capability to mediate molecular delivery through hollow microneedles. *In vivo* experiments further showcased the IEFMN's ability to track glucose fluctuations and release insulin on demand, thereby modulating blood glucose levels in diabetic rat models. The wired enzyme-coated IEFMN device was extended by coupling it with a fluidic pump system and circuit board, resulting in a wearable architecture with wireless communication functions. This development opens new possibilities for fabricating multifunctional microneedles that can seamlessly integrate with both electronic devices and fluidic systems. The IEFMN system's potential for real-time and *in situ* monitoring of diabetes, along with its intelligent and responsive diabetes management capabilities, represents a significant advancement in the field.

## Results and Discussion

**Figure 2:**
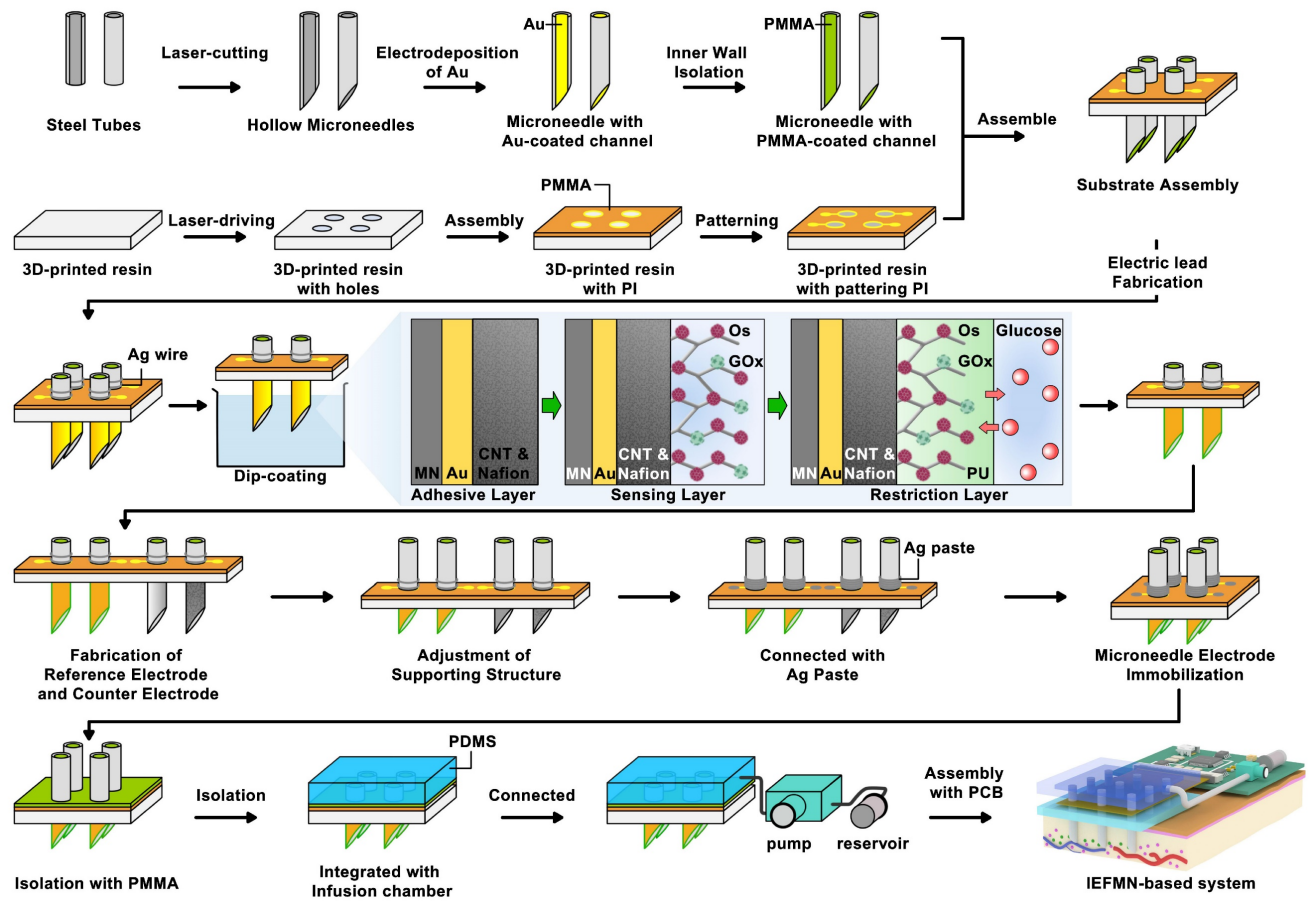
** Process flowchart for IEFMN fabrication.** (A) Hollow microneedles obtained through laser cutting of metal syringes. (B) gold plating applied to the inner walls of hollow microneedles. (C) Insulation of the inner walls of hollow microneedles with PMMA. (D) 3d-printed resin plate subjected to laser drilling (E) Pre-positioning of the 3d-printed resin plate with PI electrode pads. (F) Patterning of the PI electrode. (G) Integration of the support structure with the hollow microneedle electrode**.** (H) Hollow microneedle electrode wrapped and pre-positioned with soldered silver wire. (I) Hollow microneedle sensing electrode modification via dip coating**.** (J) Preparation of hollow microneedle counter electrode and hollow microneedle reference electrode. (K) Adjustment of the support structure and securement of the hollow microneedle electrode array using silver glue**.** (L) Insulation of the exposed electrode section with PMMA**.** (M) Integration of the hollow microneedle electrode array with the infusion chamber. (N) Connection of the infusion chamber to the peristaltic pump and reservoir. (O) Integration with the printed circuit board (PCB).

The IEFMN is constructed based on hollow microneedle electrodes that can simultaneously detect glucose fluctuations (**Figure 2**). For the fabrication of hollow microneedle electrodes, stainless steel tubes are laser-cut and oxide layer is removed by acid detergent to from hollow needles. After cleaning and drying, an Au layer is electrodeposited on the inner wall of the hollow needles to improve the electrochemical properties. Next, the inner wall of the Au-coated hollow needle is insulated using polymethylmethacrylate (PMMA) to avoid possible solution interference during usage. Meanwhile, the 3d-printed resin is laser-cut and holes are drilled into the 3d-printed resin to form a perforated substructure. Next, customized polyimide (PI) electrodes (**Figure S1**) are laminated and patterned for integration with the hollow microneedles, with silver wires attached to the wielding pads.

In the pre-position of microneedle arrays, the hollow needles are inserted into the PI electrode and the perforated substructure sequentially. The orientation and needle length are adjusted, and the position of the needles are fixed. The outer walls are then wrapped with silver wire around the hollow microneedles and glued with silver glue. The hollow microneedles inserted into the substrate had not yet adhered and fixed to the substrate, and the length of the hollow microneedles exposed to the substrate at this stage is adjustable since soldered silver wire retained sufficient length in advance. To enhance the electrode's resistance to solution, an Au layer is deposited on the outer wall of the hollow needles via electrochemical workstation to form electric leads.

In the preparation of the working electrode, Nafion solution containing Single Wall Carbon Nanotube (SWCNT) are utilized for electrochemical deposition first, constructing an adhesion layer on the outer wall of the hollow microneedle electrode. To address the weak adhesion between metal surface and macromolecules such as proteins, Nafion is introduced so SWCNT can be firmly attached to the electrode surface, which is conducive to improving the enzyme loading on the electrode surface and increasing the specific surface area of the electrode. Then, a mixture of GOx solution, Osmium mediator solution and glutaric dialdehyde solution is employed for the dip-coating of the hollow microneedle electrode to form the sensing layer. The sensing layer with a three-dimensional crosslinked mesh structure enables rapid electron transfer from the active center of the GOx to the electrode interior through the long chains of the osmium mediator, forming amperometric signals. Construction of the crosslinked mesh structure is beneficial for GOx immobilization and enzyme leakage prevention. For the construction of restriction layer, dimethylformamide solution containing polyurethane is obtained and dip-coated on the outer wall of hollow microneedle electrodes to form the working electrode. The restriction layer can restrict the glucose flux and improve the electrode biocompatibility, preventing GOx leakage from the electrode.

The hollow microneedle counter electrode is prepared by electrochemical deposition of Pt on the Au-coated hollow microneedle electrode. For the preparation of hollow microneedle reference electrodes, the outer wall of the Au-coated hollow microneedle electrodes is first uniformly coated with Ag/AgCl ink and air-dried. Then, the electrodes are dip-coated with a methanol solution containing PVB and dried at room temperature. After modification of the hollow microneedle working electrode, counter electrode, and reference electrode, the orientation, position, and needle length of each electrode are adjusted to form a hollow microneedle sensing array whose microneedles all has an exposed length of 800 μm under the support structure. Subsequently, silver wire is coated and adhered with silver glue to connect the electrodes to the wielding pads for stable electrical signal pathways. Next, a 2% PMMA solution is applied to the surface of the PI electrode, forming a hollow microneedle sensing array eventually.

**Figure 3:**
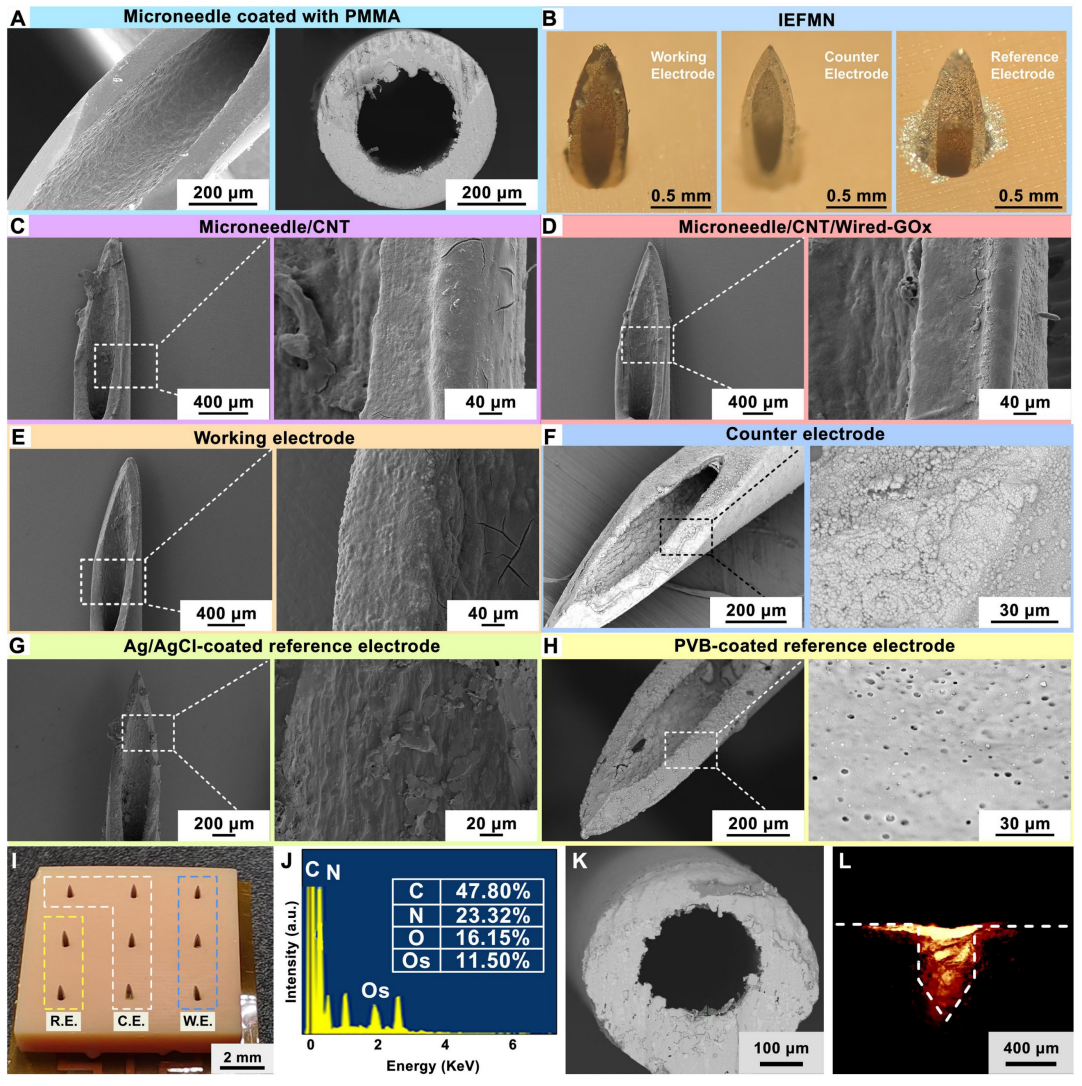
** Preparation and characterization of IEFMN electrode.** (A) Scanning electron microscope image (left) and cross-sectional scanning electron microscope image (right) depicting the microneedle exit after insulating the inner wall of the hollow microneedle with PMMA. (B) Optical view illustrating hollow microneedle electrodes, including the working electrode, counter electrode, and reference electrode. (C)-(E) Scanning electron microscope image and localized magnification revealing multi-layer structure of the hollow microneedle working electrode. (F) Scanning electron microscope image and localized magnification of the hollow microneedle counter electrode. (G)-(H) Scanning electron microscope image of different structure on hollow microneedle reference electrode (with localized magnification). (I) Optical view illustrating the array of hollow microneedles. (J) Energy-dispersive X-ray spectroscopy (EDS) analysis of the sensing layer of the hollow microneedle working electrode. (K) Cross-sectional scanning electron microscope image showing the inner wall of working electrode after PMMA insulation. (L) Fluorescence image showing rhodamine B deposition into porcine skin after IEFMN penetration.

The surface morphology of the hollow microneedle electrodes during fabrication are characterized using scanning electron microscopy (SEM). As shown in **Figure 3A**, after insulating the hollow microneedle channels with PMMA, a uniform layer of PMMA is observed adhering to the inner walls of the hollow microneedle channels at the exit, without causing any blockage. **Figure S2A-B** suggests that as the PMMA concentration increased, the insulation layer thickness increased and a well-defined thickness is achieved at 2% PMMA concentration. To validate the permeability of the PMMA-modified inner walls of the hollow microneedle channels, the microneedle walls are cross-sectioned using laser cutting and observed under scanning electron microscope. The cross-sections are uniform, and a thin layer of PMMA is observed on the inner walls of the hollow microneedle, with no signs of blockage in the channels. For the hollow microneedle electrode array, a 3×3 array layout, including 3 glucose sensing electrodes, 4 counter electrodes, and 2 reference electrodes is designed. As showed in **Figure 3B**, the hollow microneedle sensing electrodes are vertically arranged on the substrate, with a length of approximately 800 μm above the substrate. The outer diameter of the hollow microneedles is 450 μm, and the inner diameter is approximately 230 μm. The surface structure of the hollow microneedle glucose sensing electrodes is characterized using SEM. For the hollow microneedle glucose sensing electrodes, a composite coating of osmium mediator and GOx is attached to the microneedle surface, resulting in a rough surface morphology, as shown in **Figure 3C-E**, without a significant increase in microneedle outer diameter.

Regarding the hollow microneedle counter electrodes, the electrode surface displayed a layer of dense platinum particles, with an average particle size of approximately 500 nm, as observed in the magnified structure image (**Figure 3F**). As for the hollow microneedle reference electrodes, a uniform PVB protective layer is observed on the electrode surface (**Figure 3G-H**), with sporadic distribution of pores ranging from 1 μm to 3 μm, corresponding to the colloidal structure of PVB. As shown in **Figure 3I**, a 3x3 array of 9 electrodes is placed on the support structure with a 3 mm distance between needles. Elemental analysis of the hollow microneedle glucose sensing electrode surfaces using energy-dispersive X-ray spectroscopy (EDX, Wavetest) revealed the presence of prominent Os peaks (~2 keV) in the EDS spectra, indicating successful modification of osmium mediator on the surface of the hollow microneedle sensing electrodes (**Figure 3J and Figure S2C**). The sensor-coated hollow microneedle electrodes are cross-sectioned using laser cutting, revealing a thin layer of PMMA on the inner walls of the hollow microneedles, as observed under a scanning electron microscope, without causing blockage to the microneedle channels (**Figure 3K**).

Characterization of the hollow microneedle electrode in penetrating the skin is carried out next. Fresh porcine skin is employed to simulate human skin, and red fluorescent dye is applied to the microneedle tip for staining. Subsequently, the hollow microneedle electrode is connected to a mechanical fixture and placed vertically above the fresh porcine skin surface. Vertical pressure is applied downward to insert the hollow microneedle electrode tip through the porcine skin surface. After the microneedle tip penetrated the skin, it is left in place for 20 s before removal. Using a surgical blade, further observation is performed by obtaining approximately 200 μm thick porcine skin along the cross-sectioned holes left by microneedle to evaluate the skin puncturing performance of the microneedle. These sections are placed on glass slides and observed under a fluorescence microscope to examine the deposition of the red fluorescent dye. As shown in **Figure 3L**, the red fluorescent dye is clearly visible in the cross-section of the porcine skin, emitting red fluorescence around the penetration site of the hollow microneedle. These results consistent in shape with the morphology of the hollow microneedle electrode's tip, confirming the effective skin penetration capability of the hollow microneedle electrode's tip. Furthermore, mechanical tests are conducted to assess the penetrating ability of the hollow microneedle into fresh porcine skin. First, the hollow microneedle is vertically positioned in a mechanical test fixture and secured, while fresh porcine skin is placed on foam. The fixture is software-controlled to move downward slowly and uniformly, ensuring that the hollow microneedle tip pierced the skin vertically and stopping when the microneedle tip reached maximum depth. After remaining on the skin for 20 seconds, the fixture is slowly moved upward at the same speed, retracting the hollow microneedle tip from the skin along the original path and recording the force exerted during the insertion and withdrawal processes using a mechanical force gauge. The results suggest that the force gradually increased during the insertion of the hollow microneedle into the skin, with a puncture force of approximately 0.53 N (**Figure S3A**). These results indicate that within the range of normal pressing forces, the hollow microneedle tip is capable of effectively penetrating the skin, aided by its sharp structural design.

**Figure 4:**
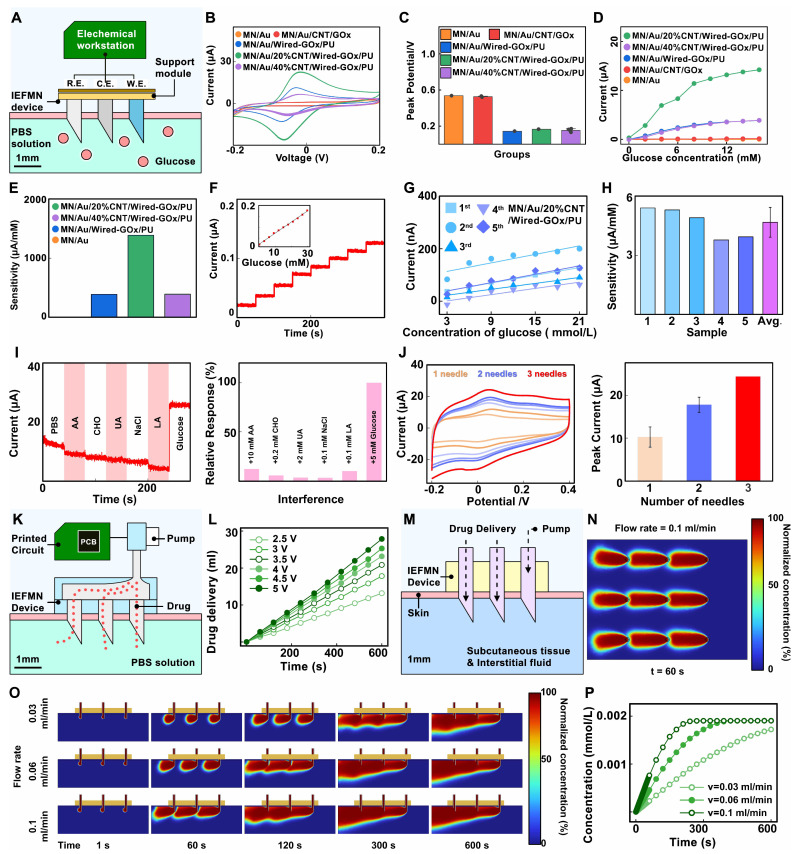
** In-vitro characterization of IEFMN electrode.** (A) Schematic outlining the in-vitro electrochemical testing of IEFMN. Cyclic voltage curves (B) and peak position statistics (C) of the working electrode with varying component ratio doping in the sensing layer. (D) Continuous current response and (E) sensitivity analysis of the hollow microneedle working electrode with different component ratio in varying glucose concentrations. (F) Continuous current response of the hollow microneedle working electrode to varying glucose concentrations. (G)-(H) Parallel experimentation of the hollow microneedle working electrode under different glucose concentrations. (I) Specific responses of the hollow microneedle working electrode to glucose with relative changes in the response current (right, with absolute values). (J) Cyclical voltammetry curves of the hollow microneedle working electrode incorporating multiple microneedles with peak positions statistical presentation (right). (K) Schematic diagram showing drug delivery experiments of the IEFMN connected with the supporting circuit. (L) Delivery amount within 10 mins using IEFMN in different excitation voltage. (M) Schematic showing the model setup of the IEFMN for drug delivery *in vitro*. The insulin concentration distribution including top view (N), screenshots (O) and total delivered amounts with different flow rates (P) within 10 mins.

Next, the electrochemical performance of the fabricated hollow microneedle glucose electrode is characterized. As illustrated in **Figure 4A**, a 25 mL beaker containing 20 mL PBS phosphate buffer solution is prepared for the simulation of interstitial fluid, covered with a 4-folded parafilm film to emulate *in vivo* conditions. The hollow microneedle electrode is pierced through the film to simulate transdermal penetration, and the electrode tip is immersed in the testing solution to simulate detection within interstitial fluid. Employing the hollow microneedle sensor electrode as the working electrode, a platinum electrode as the counter electrode, and a commercial Ag/AgCl electrode as the reference electrode, the electrochemical performance of the wired enzyme hollow microneedle sensor electrode is measured via a CHI760E workstation. Initially, cyclic voltammograms are recorded in the range of -0.2 V to 0.4 V (scan rate: 0.1 V/s) to locate the amperometric peak position. To validate the effect of wired-GOx in reducing the operating voltage, cyclic voltammetry is conducted on the MN/Au electrode, MN/Au/20%CNT/Wired-GOx/PU electrode, MN/Au/40% CNT/Wired-GOx/PU electrode and the MN/Au/CNT/Wired-GOx/PU electrode. As shown in **Figure 4B and Figure S4,** for the MN/Au electrode, a current peak appeared at 0.53 V, with a current magnitude of approximately 2.0 μA. As for the MN/Au/CNT/GOx electrode, a current peak appeared at 0.53 V, with a current magnitude of approximately 2.8 μA. For the MN/Au/Wired-GOx/PU electrode, a current peak emerged at 0.14 V, with a current magnitude of about 11.36 μA. As for the MN/Au/20%CNT/Wired-GOx/PU electrode, a peak is evident around 0.16 V, accompanied by a current magnitude of roughly 22.8 μA. Similarly, for the MN/Au/40% CNT/Wired-GOx/PU electrode, a peak occurred at 0.14 V, with a current magnitude of approximately 6.65 μA. Statistical analysis on the peak currents of different kinds of electrodes is further conducted to observe the current peak directly (**Figure 4C**). The peak position in the voltammogram of the hollow microneedle electrode without wired-GOx is approximately 0.5 V. In contrast, hollow microneedle electrodes modified with wired-GOx displayed a peak position around 0.15 V in the voltammogram, far lower than electrodes mentioned above. The results demonstrated that the introduction of wired-GOx distinctly shifted the amperometric peak towards lower voltages compared to pure gold electrodes, affirming the voltage reduction effect of wired-GOx. Notably, with the addition of carbon nanotube, the charge-carrying capacity of the electrode increased, resulting in amplified peak currents and an increased area enveloped by the cyclic voltammogram curve. Meanwhile, the current peak remained relatively consistent, indicating that the introduction of carbon nanotube materials did not interfere with the mechanism of voltage reduction by wired-GOx.

Through the experiments, the detection voltage of the electrode modified with wired-GOx is determined to be around 0.15 V. Amperometric i-t curve is further collected by gradually increasing the glucose solution concentration from 0 mmol/L to 16 mmol/L and applying a certain bias voltage (0.15 V vs Ag/AgCl electrode) on the surface of the hollow microneedle sensor electrode. According to **Figure 4D-E**, for the MN/Au electrode and the MN/Au/CNT/GOx electrode, the current magnitude remained virtually unchanged as the glucose concentration rose from 0 mmol/L to 16 mmol/L. This is primarily attributed to the weak conductivity of the electrode under low voltage, which hindered the effective transfer of electrons from enzymes to the gold layer of the electrode. In comparison, the MN/Au/Wired-GOx/PU electrode registered an incremental current surge from 1 nA to 3.86 μA as glucose concentration rose, resulting in an average sensitivity of 386.7 nA/mM. For the MN/Au/20%CNT/Wired-GOx/PU electrode, the registered current swelled from 287 nA to 14.21 μA, translating to an average sensitivity of 1.39 μA/mM. Correspondingly, the MN/Au/40% CNT/Wired-GOx/PU electrode witnessed an ascending current from 0 nA to 3.88 μA, yielding an average sensitivity of 0.39 μA/mM.

The results above indicate that the addition of a carbon nanotube coating to wired-GOx significantly enhances the response sensitivity of the hollow microneedle sensor electrode to glucose. This phenomenon can be attributed to the favorable nano-network structure of nanotubes, which enhances cross-linked contacts with osmium mediator. Upon the transfer of redox electrons from enzymes to the osmium mediator, a more efficient transfer to carbon nanotubes and subsequently to the gold layer of the electrode is facilitated. Notably, the MN/Au/40% CNT/Wired-GOx/PU electrode does not exhibit exceptional capacitance ability in cyclic voltammetry, and the amperometric signal of i-t curve is not substantial. Excessive doping ratios of CNT may affect the surface structure of the electrode, thereby reducing the stability of the electrode during use. Therefore, the MN/Au/20%CNT/Wired-GOx/PU electrode is chosen as the working electrode for IEFMN.

Next, the selectivity of the IEFMN is assessed using an electrochemical workstation. Selectivity performance is evaluated by applying a bias voltage of 0.15 V vs Ag/AgCl to the electrodes in an *in vitro* simulated solution, gradually increasing the glucose concentration from 3 mmol/L to 21 mmol/L. Amperometric signals are collected to assess the electrochemical performance of the hollow microneedle glucose sensing electrode. Facilitated by the redox reactions of ruthenium ions, the immobilized GOx on the surface of the electrode selectively catalyzes the oxidation of glucose in the solution, leading to the generation of gluconic acid lactone. The electrons produced during the redox reactions are transferred through the wired-GOx to the carbon nanotubes and electrode layer. Consequently, an efficient electronic transfer occurs from the enzyme to the electrode, and the resulting electrical signal is transmitted across the electrode surface to the internal electrode, forming an amperometric circuit. The transformation of glucose in the solution results in the generation of a measurable amperometric signal, thus converting the previously unquantifiable glucose concentration into an interpretable current response. The outcomes demonstrate that as the glucose concentration incrementally rises from 0 mmol/L to 21 mmol/L, the response signal escalates from 20 nA to 126 nA. The linear fitting of the current signals against glucose concentration illustrates a robust linear relationship, with a sensitivity of 4.06 μA/mM and a linear correlation coefficient (R^2^=0.99, **Figure 4F**). The difference between this and the previous results may be due to individual differences in electrodes or environmental factors. These results collectively underscore the commendable responsiveness of the hollow microneedle glucose sensing electrode to glucose fluctuations within the concentration range of 0 to 21 mmol/L. Furthermore, an evaluation of the performance reproducibility of the batch-prepared hollow microneedle glucose electrodes is conducted. Five distinct hollow microneedle glucose electrodes from the same batch are placed to varying concentrations of glucose to evaluate their responses. The results reveal analogous trends among these batch-prepared electrodes as glucose concentration increases, with glucose detection sensitivities of 3.78 μA/mM, 3.94 μA/mM, 4.91 μA/mM, 5.31 μA/mM, and 5.41 μA/mM, respectively. The average sensitivity is calculated to be 4.68 μA/mM, and the relative deviation in electrode sensitivities is 16.5% (**Figure 4G-H**). Changes in sensor sensitivity can be caused by environmental factors during storage or repetitive testing, which can be reduced by optimizing the modification process and test conditions.

Meanwhile, selective testing is conducted to validate the specific glucose detection capability of the hollow microneedle glucose sensing electrode. A sequential addition of 10 mmol/L ascorbic acid, 0.2 mmol/L cholesterol, 2 mmol/L uric acid, 0.1 mmol/L sodium chloride (representative of salts solution), 0.1 mmol/L lactic acid, and 5 mmol/L glucose is performed in a phosphate-buffered saline (PBS) solution while recording the amperometric signals of the electrode within 300 s. As showed in **Figure 4I**, upon glucose addition, the current increased from 5 μA to 30 μA. However, the introduction of interferents such as lactic acid and potassium chloride resulted in variations of no more than 2.5 μA, demonstrating that the presence of interferents does not impact the glucose detection capability of the hollow microneedle glucose sensing electrode. Furthermore, to visually demonstrate the specificity of the hollow microneedle glucose sensing electrode, an analysis of signal changes generated by the electrode is carried out upon introduction of different substances. For comparative purposes, the signals change upon addition of glucose solution is set as 100%, and the signal changes caused by various interferents are normalized. Additionally, the relative interferences caused by substances such as lactic acid and potassium chloride are 12%, 5%, 3%, 3%, and 10%, respectively, compared to glucose. These experiments indicate that the current interference caused by substances are no more than 13% relative to glucose, demonstrating that the hollow microneedle glucose sensing electrode maintains stable respond to glucose concentration changes even in the presence of lactate, salts, cholesterol, ascorbic acid, and uric acid.

In response to the challenge of low sensitivity due to the limited sensing area of the hollow microneedle electrode, a multi-electrode detection approach is proposed. the electrochemical performance of the series-connected hollow microneedle glucose sensing electrodes is validated by employing an electrochemical workstation to perform cyclic voltammetry tests in a phosphate-buffered saline (PBS) solution on single, double, and triple hollow microneedle electrode configurations. **Figure 4J** demonstrated that the peak voltage for a single microneedle electrode occurred at 0.08 V with a peak current of approximately 10.1 μA. For the dual microneedle electrode, the peak voltage is observed at 0.09 V with a peak current of around 18.3 μA. As for the triple microneedle electrode, the peak voltage is recorded at 0.08 V with a peak current of approximately 24.1 μA. These experimental findings validate that increasing the number of parallel-connected hollow microneedle electrodes effectively enhances the response signal amplitude and detection sensitivity of the glucose sensing electrode, thus further augmenting the resistance to interference signals in the hollow microneedle sensing array. In the construction of the hollow microneedle sensor, a series connection of three hollow microneedle glucose sensing electrodes is chosen, following a ratio of working electrode to counter electrode to reference electrode of 3:4:2.

Next, to validate the drug delivery performance of IEFMN, a multilayer parafilm is utilized to simulate the human skin stratum corneum, and the film is punctured by the IEFMN to reach the container space underneath the film. A circuit board is utilized to control the peristaltic pump to drive the solution flow in the IEFMN, delivering the aqueous solution from the reservoir through the IEFMN to the container underneath the parafilm (**Figure 4K**). The volume of aqueous solution delivered by the IEFMN are measured with the delivery time at different drive voltages (2.5 V to 5 V). It is found that the volume of the IEFMN-delivered solution increased linearly with time, and the increase of the driving voltage also significantly enhanced the volume of the IEFMN-delivered solution (**Figure 4L and Figure S3B**). A statistical analysis of the flow rate of the solution delivered by the IEFMN is performed, suggesting that the flow rate of the IEFMN gradually increased from 1.42 ml/min to 2.98 ml/min as the excitation voltage is gradually increased from 2.5 V to 5 V. These results indicated that the hollow tube of the IEFMN can effectively deliver the solution with the support of a peristaltic pump, and that the solution delivery flow rate can be precisely controlled by changing the excitation voltage of the control circuit. The drug delivery process of the IEFMN in 3D are simulated using the Dilute Matter Delivery and Laminar Flow modules of COMSOL simulation software (**Figure 4M and Figure S5-S7**). The geometry and dimensions of the 3 x 3 microneedle device is referenced, with the stratum corneum and epidermis of the skin modeled, separately. The IEFMN is inserted into the skin, and the dorsal part of the IEFMN is connected to the device for drug delivery. A solution containing a macromolecule (molecular weight 6,000, simulating an insulin molecule) is set up at a concentration of C_0c_= 1 mol/m^3^ at the dorsal border of the IEFMN and delivered it through the IEFMN at a set flow rate into the skin tissue beneath it.

To observe the distribution of insulin in the subcutaneous tissue, the spatial distribution of the drug delivered into the skin tissue through the IEFMN are calculated, and the average concentration of the molecule accumulated in the skin tissue are further calculated. **Figure 4N** shows the molecular concentration distribution in the xy-directional profile of the skin tissue after 60 s delivery. The dynamic diffusion process of IEFMN delivery of macromolecular solutions into skin tissues under different flow rate (0.03 ml/min, 0.06 ml/min, 0.1 ml/min) conditions are calculated (**Figure 4O**). The results suggest that the IEFMN-delivered molecules gradually penetrated the subcutaneous tissue layer with increasing time, and the molecular diffusion gradually leveled off after 300 s. As the flow rate increased, the cumulative concentration of delivered molecules in the skin tissue increased significantly and can reach deeper into the subcutaneous tissue. As showed in **Figure 4P**, the dynamic accumulation of macromolecules delivered by IEFMN under different flow rates are further statistically analyzed. The concentration of IEFMN-delivered molecules in the subcutaneous tissue below the IEFMN increased linearly with time and finally reached saturation (~0.002 mmol/L). At a flow rate of 0.1 ml/min, the cumulative subcutaneous concentration of IEFMN-delivered molecules saturated at ~300 s, while at flow rates of 0.06 ml/min and at 0.03 ml/min, the cumulative subcutaneous concentration of IEFMN-delivered molecules saturated after approximately 400 s and 600 s. The IEFMN device is designed for the delivery molecules can be continuously replenished into the reservoir on the back of the device, suggesting that the IEFMN device had a good drug loading capacity and could be used in combination with a regulated peristaltic pump flow rate to control the drug delivery volume.

**Figure 5:**
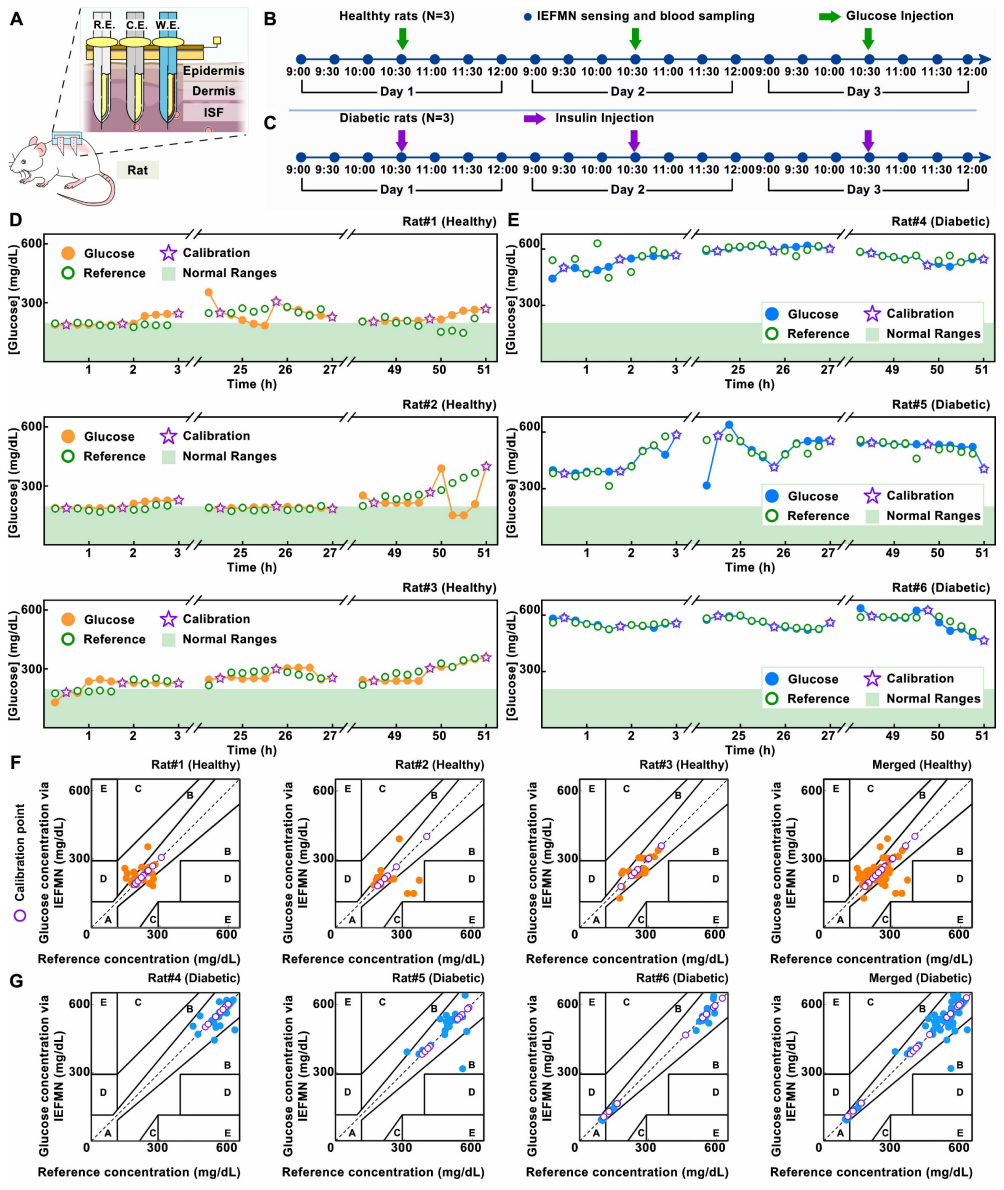
** Performance characterization of IEFMN *in vivo*.** (A) Schematic diagram showing the simultaneous applications of the IEFMN on a rat. Timeline of the 3 h-detection experiment in 3 days for (B)healthy rats and (C) diabetic rats. (D) The dynamic glucose signals measured by IEFMN and standard reference methods on 3 healthy rat samples individually. (E) The dynamic glucose signals measured by IEFMN and standard reference methods on 3 diabetic rat samples with Clarke's error grid analysis individually. (F)-(G) Clarke's error grid analysis showing the detection accuracy of glucose by IEFMN, compared to the actual BGLs measured via standard glucometer. The reference concentrations of glucose in the blood of rats are measured at certain time points using a commercial glucose meter, and the corresponding Clark analysis. using a commercial glucose meter, and the sensing results of the microneedle sensor are calibrated according to the reference concentrations (purple stars in the plots).

Next, the feasibility of IEFMN in detecting blood glucose fluctuations *in vivo* are verified on rats (**Figure 5A**). The animal experiments included both healthy and diabetic rats, with each group consisting of 3 rats as parallel controls. After anesthetizing and depilating the rats, IEFMN is utilized to penetrate the skin on the rat's back, ensuring close contact and epidermal penetration. The experiment commenced with the recording of amperometric measurements by IEFMN every 15 minutes, continuing for a duration of 3 hours for each microneedle glucose monitoring trial. Based on the current-glucose concentration standard curve obtained from *in vitro* testing, the signals collected are converted into glucose concentrations. Blood samples are collected every 15 minutes from the rat's tail artery, and commercial blood glucose meters are used to measure glucose levels in the rat's blood as a control. For the healthy rats, this study induced blood glucose fluctuations by subcutaneously injecting 3 mL of 5% glucose solution (w/v) at the 90-minute mark using a syringe (**Figure 5B**). For the diabetic rats, insulin solution (1.2 mL, approximately 8 IU) is subcutaneously injected at the 90-minute mark (**Figure 5C**). The animal experiments are repeated over 3 days, and for each day's experiment, the same procedures are repeated. IEFMN is used to measure tissue fluid glucose concentrations in the rats, while commercial blood glucose meters are used to verify blood glucose levels from blood samples as reference. Considering the variances between *in vitro* and *in vivo* environments, a conversion equation between sensor signals and glucose concentration is derived, utilizing amperometric signals from microneedles and standard blood glucose measurements via a glucometer. To ensure stable signals at the onset of *in vivo* experiments, the standard curve is established by aligning current signals from the second measurement point (30 minutes) and the last point (3 hours) with corresponding blood glucose values each day. Due to potential disturbances stemming from insulin or glucose administration at the 90-minute mark and its potential impact on anesthetized rats, the current signals at the 105-minute mark are employed to fine-tune the *in vivo* experiment's standard curve.

Furthermore, accuracy of IEFMN in detecting intertrial glucose level of live animals is analyzed. For each rat, the fluctuations in blood glucose concentrations measured by the IEFMN and standard blood glucose values by a commercial instrument over 3 days are compared. As shown in **Figure 5D-G**, the orange or blue dots represent the blood glucose values measured by IEFMN in healthy and diabetic rats, respectively, while the green dots represent the reference blood glucose concentrations. The purple stars indicate the blood glucose concentrations used for establishing the standard curve or calibration, and the light green band represents the normal range of blood glucose levels in healthy bodies. The trends of glucose fluctuations measured by the microneedle closely matched those of standard blood glucose fluctuations for each rat. In healthy rats, the blood glucose levels increased for all groups after the glucose injection. For diabetic rats, the decrease in blood glucose levels after insulin injection is not prominent, primarily due to the severe diabetic conditions of the rats, where a single subcutaneous insulin injection did not significantly reduce blood glucose levels.

**Figure 6:**
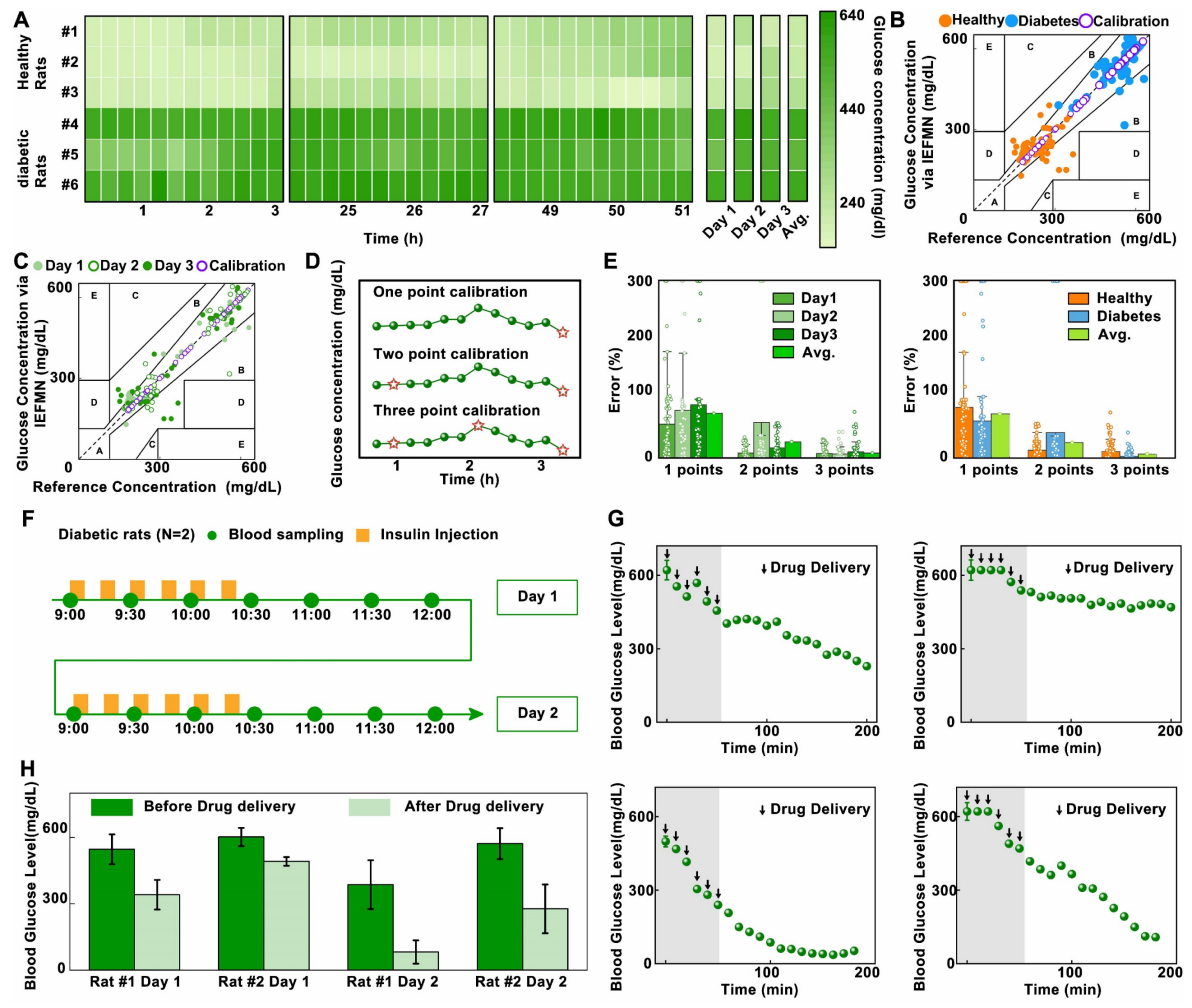
** Analysis of IEFMN for long-term *in vivo* application.** (A) The heatmap plots of dynamic fluctuation of glucose concentrations measured via IEFMN on 2 experimental groups (each group employed 3 rats in parallel). (B) Clarke's error grid analysis showing the detection accuracy of glucose by IEFMN on 2 experimental groups, compared to the actual BGLs measured via standard glucometer. The error range for each region is shown in the figure. (C) Clark's error grid analysis showing the daily detection accuracy of glucose by IEFMN, compared to the actual BGLs measured via standard glucometer. (D) Comparison of calibration points (orange stars) adopted by different calibration methods. (E) Statistical analysis showing the detection error of the IEFMN when different calibration methods are applied. N = 3 samples. (F) Timeline of the 2-day drug delivery experiment using IEFMN. (G) The dynamics of blood glucose in 2 rats during the 2-day drug delivery experiment via IEFMN. (H) Statistical analysis of the change in glucose concentration (ΔGlucose) in rats during the 2-day drug delivery, N = 2 samples.

Additionally, the blood glucose fluctuations of rats (2 groups) over 3 days are summarized and compared using a heatmap representation (**Figure 6A**). The average daily blood glucose concentration and the mean values of blood glucose concentration per rat in both healthy and diabetic rats are summarized, respectively (**Figure S8-S9**). During this period, the average blood glucose concentrations of three healthy rats are 216.5 mg/dL, 217.5 mg/dL, and 257.8 mg/dL, respectively. For diabetic rats, the average blood glucose concentrations are 563.7 mg/dL, 487.8 mg/dL, and 560.8 mg/dL. This is attributed to the utilization of three reference points for calibrating the standard curve between the sensor signal and blood glucose concentration, alongside the implementation of an additional blood reference for data correction.

At the same time, further analysis of the IEFMN detection accuracy is performed using Clarke error grid. The results are presented with respect to the categories of healthy and diabetic rat models (**Figure 6B**) as well as the experimental days (**Figure 6C**). For both healthy and diabetic rats, most of the daily sensor-detected data points fall within zones A and B, indicating that the overall measurement errors are below 20%. The distribution of detected glucose values in healthy rats falls within the moderate glucose range, while in the diabetic rat model, the detected glucose values are distributed in the high glucose range. The comparable dispersion of microneedle glucose detection accuracy between these two models suggests that the detection range of IEFMN can cover the glucose fluctuation range in both diabetic and healthy rats. When categorized by experimental days, the distribution of microneedle glucose detection accuracy on the first, second, and third days is relatively uniform, with no significant differences observed in accuracy between each day. Error of glucose detection by IEFMN for each rat suggests that most data points are situated within a region characterized by errors of less than 15% (**Figure S10**). The detection error for diabetic rats is marginally lower than that observed in healthy rats, possibly owing to the elevated blood glucose levels inherent to diabetic rats.

To further validate the enhancement effect of the multi-points calibration on the accuracy of IEFMN *in vivo* glucose detection, statistical analysis of measurement errors with different calibration is conducted (**Figure 6D and Figure S11**). As showed in **Figure 6E**, categorized by experiment dates, the results indicate that under single-point calibration, the average daily error is close to 80%, reaching a maximum of 93%. Under two-point calibration, the average daily error is around 50%, reaching a maximum of 80%. Under three-point calibration, the average daily error remained within 18%. Categorized according to rat models, the results indicated that under single-point calibration, the average detection error for both groups and the entire dataset is close to 80%. Under two-point calibration, the average detection error for each group and the entire dataset is close to 30%. Under three-point calibration, the average detection error for both rat groups and the entire dataset remained within 15%. These findings demonstrate that for the daily blood glucose detection results of both healthy and diabetic rats, the statistical error associated with the three-point calibration method is smaller than that of the single-point or two-point calibration methods. The three-point calibration method exhibits higher accuracy in the *in vivo* glucose detection of IEFMN. Increasing the number of calibration points contributes to improving the blood glucose detection accuracy. However, excessive calibration points may increase operational complexity and patient burden in practical applications. Therefore, the choice of calibration points should strike a balance between maintaining reasonable accuracy and minimizing complexity.

Next, to validate the transdermal drug delivery capability of the hollow microneedle array, *in vivo* experiments are conducted to assess the performance of IEFMN in drug delivery. The animal experiments included 2 diabetic rats. After anesthesia and depilation, the rats are subjected to procedures involving the usage of a hollow microneedle array to penetrate the skin from their dorsal region, ensuring sufficient contact with the skin and penetrated epidermally. At fixed time intervals (every 15 minutes), the hollow microneedle array is employed to deliver equal amounts of insulin solution (0.8 mL, approximately 0.2 IU, Qiyun Biotechnology Co., Ltd., Guangzhou) to the rats, with a total of 6 deliveries. The insulin solution could be introduced into the rats' subcutaneous tissue through the internal channels of the hollow microneedle array. Blood samples are collected from the rats' tail artery every 10 minutes, and the blood glucose levels are measured using a commercial blood glucose meter. The experiment lasted for about 3.5 hours. The animal experiments are repeated the next day, and the changes in blood glucose levels are monitored (**Figure 6F**).

Next, analysis and statistical evaluation blood fluctuation of rats over time are conducted. The initial rat blood glucose levels are high, exceeding the saturation value of commercial instrument (600 mg/dL). The results demonstrate that, the blood glucose levels of the rats gradually decreased from >600 mg/dL to an overall average of approximately 300 mg/dL, with a maximum decrease to as low as 20 mg/dL over time (**Figure 6G**). This is attributed to the increase of insulin amount delivered by the hollow microneedle array as time extended. The elevated insulin concentration in the rats' bodies accelerated consumption rates of glucose, leading to a pronounced downward trend in blood glucose levels.

Subsequently, a comparison and analysis are carried out on the average blood glucose levels of the model rats before and after microneedle drug delivery. As showed in **Figure 6H**, the reductions in blood glucose levels for Rat 1 after delivery on the first and second days of the experiment are approximately 200 mg/dL and 305 mg/dL, respectively. For Rat 2, the reductions in blood glucose levels after insulin delivery in the two-day experiment are approximately 108 mg/dL and 300 mg/dL, respectively. Results above suggest that the hollow microneedle array is capable of sustained release of insulin into the subcutaneous tissue fluid of model rats, significantly lowering blood glucose levels in diabetic rats, thus exhibiting the ability for *in vivo* blood glucose regulation.

**Figure 7:**
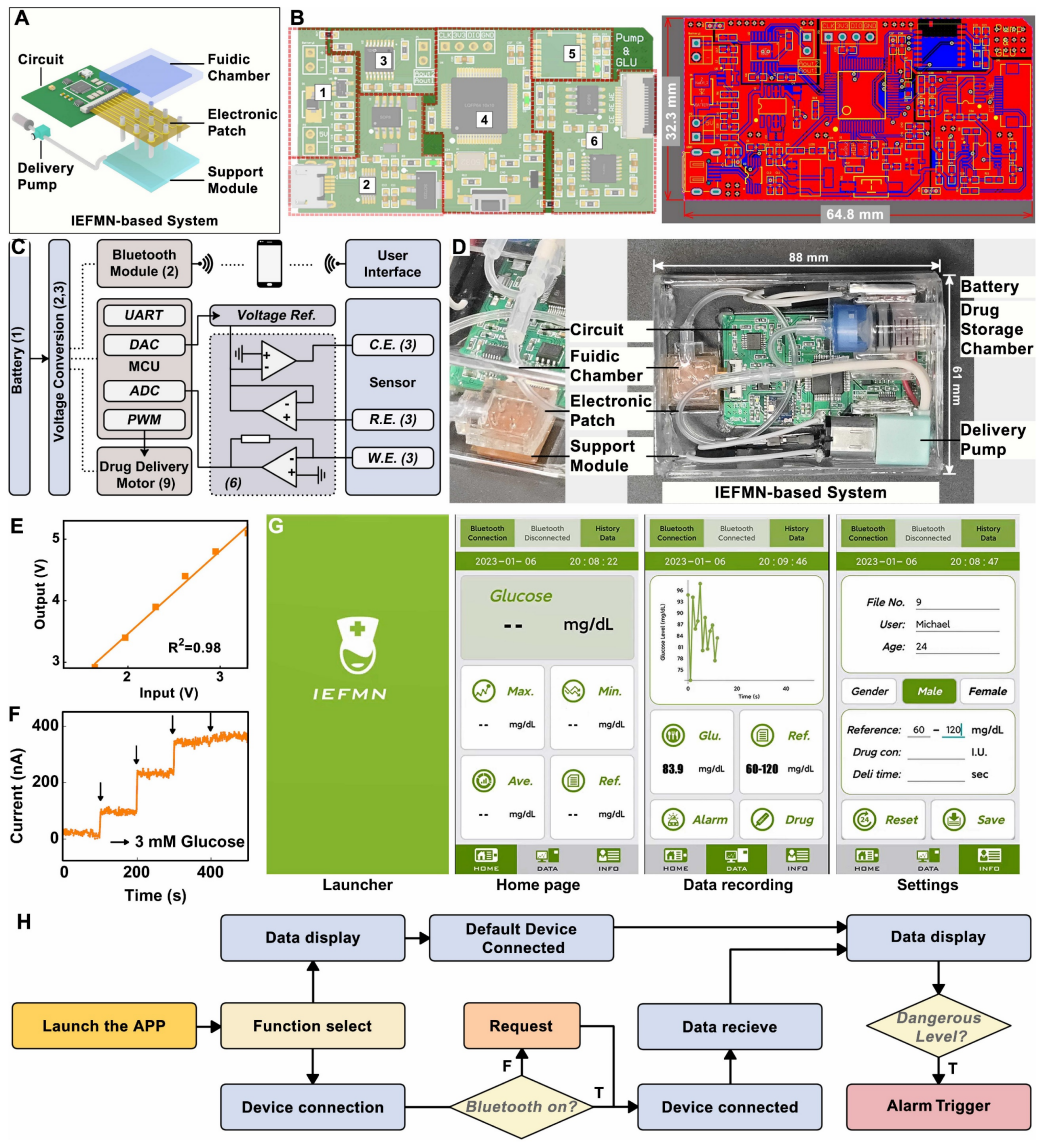
** Circuit design and characterization of IEFMN.** (A) Schematic view of the IEFMN-based system and its components. (B) Design sketch (left) and detailed components of a PCB (right). (C) The system block diagram of PCB. (D) The photograph of an IEFMN. (E) Input-output signal calibration for glucose sensing signal channel. (F) *In vitro* electrochemical performance of IEFMN. (G) The launcher page, home page, data recording and setting page of the smartphone APP. (H) Function logic schematic of the IEFMN application.

For the integration of the IEFMN-based blood glucose monitoring and regulation system (**Figure 7A**), an embedded system to assist the IEFMN for glucose detection and drug delivery is developed (**Figure 7B**). Details of the components of each module are given in **Figure S12-S18**. The embedded wearable circuit is equipped with functions including signal conditioning, processing, and wireless transmission, which help to realize the integration and miniaturization of the IEFMN (**Figure 7C**). The IEFMN is connected to the embedded circuit through a polyimide electrode interface, whereas the peristaltic pump is connected to the embedded circuit via DuPont wires. The embedded circuit is designed to contain not only the detection module and drug delivery module, which are used to support both electrochemical detection of glucose detection and peristaltic pump for drug delivery, respectively, but also a high glucose warning function.

During detection, analog signals are processed and transmitted to the microcontroller, before being converted to digital signals through the microcontroller's ADC port. The electrochemical module ensures the stable recording of the amperometric signal and glucose concentration track through time-division multiplexing technology. The data output from the microcontroller can be transmitted to a smartphone application via a wireless transceiver for real-time observation. The IEFMN-based blood glucose sensing and regulation system (dimensions 88 × 61 × 54 mm, weight 54.8 g) is integrated by connecting the IEFMN and embedded circuitry, with a built-in 5 V power supply to support the microneedle device for glucose fluctuations monitoring and tracking, working as a wearable device (**Figure 7D**).

Meanwhile, linearity of the embedded circuit in signal input and output is characterized. The designed and prepared detection circuit generates an amperometric signal at a constant voltage from 1.6 to 3.3 V and converts them to the corresponding voltage signal by means of an AD5220 resistor chip, i.e., recording the voltage signal and obtaining the corresponding output. As showed in **Figure 7E**, after voltage signal input, the analog electrochemical signal output from the circuit system shows a good linear relationship with the input voltage signal (R^2^ = 0.98), indicating that the stable input voltage and stable electrical signal output of the circuit system can be used to support electrochemical sensing of IEFMN.

The circuit is employed to collect amperometric signals from IEFMN to detect glucose concentration under conditions where the glucose concentration varied from 0 to 12 mmol/L. Using the prepared circuit system connected to the IEFMN, the acquired signals increased with the concentration of glucose solution. **Figure 7F** suggests that the acquired signals which are consistent with the results obtained via electrochemical workstation, both exhibited a similar trend with the increase in glucose concentration. The smartphone application, shown in **Figure 7G**, consists of a main interface displaying the average glucose level and hyperglycemia warning, and a sub-interface displaying the glucose level over time. This module is capable of real-time monitoring and fluctuations in glucose levels with early warning and drug delivery (**Figure 7H and Figure S19**), suggesting that the circuit and app can be used to support the operation of glucose sensing and drug delivery device. In addition, the signal accuracy of the inputs and outputs of the wearable circuit system can be linearly calibrated with software to eliminate non-sensor-induced errors like current drift or interference from foreign objects, thus improving the sensor stability. The above results show that the embedded system developed can provide effective voltage outputs and performing signal acquisition to assist in the realization of the IEFMN sensing function.

## Conclusion

In this work, an IEFMN patch-based system is designed and constructed for blood glucose monitoring and regulation. The IEFMN, developed through a dimensional reduction process, integrates a sensing module and a drug delivery module on the same hollow microneedle, achieving continuously glucose monitoring as well as transdermal insulin delivery, which solves the problem of multifunctional sensor manufacturing. Based on the introduction of wired enzyme and carbon nanotube, the IEFMN possesses high sensitivity, excellent selectivity, and a wide response range (up to 30 mM) under lower voltage (0.14 V vs Ag/AgCl), avoiding susceptibility to external and internal interference. The as-developed system offers painless and minimally invasive penetration via microneedle array, along with reduced sensing potential through wired enzyme functionalization, which enhances the accuracy of *in vivo* glucose measurements. Through coupling the hollow microneedle sensing array with micro-channel module and embedded circuits, the system is capable of percutaneous drug delivery, as well as data collection, tracking, and display through a wearable and wireless design. This contributes to real-time, intelligent, and controllable diabetes management. It possessed potential opportunities for advancing microneedle substrate glucose sensors, integrating microneedle electronic systems, and developing intelligent diabetes diagnosis and treatment systems.

## Materials and Methods

***Microneedles tip fabrication and modification:*** Stainless steel syringes are employed as the substrate material for microneedle electrodes. YLP-F Series Optical Fiber Laser marking Machine are utilized (Han's Laser Technology Industry Group Co., Ltd.) to cut the SUS304 stainless steel syringe into hollow needles with a length of 6 mm. The conditions are as followed: Laser wavelength 1.06 μ m, engraving line speed 1500 mm/s, power 18 W, and engraving 1200 times. The hollow syringes were soaked in acid detergent (Yuncai Taotao Co., Ltd.) to remove the oxidized layer on the surface of the hollow syringes, which were then washed with ethanol and dried in an oven (80 degrees C). Next, an Au layer is electrodeposited on the inner wall of the hollow needles to improve the electrochemical properties. With the Au electrode as the anode, the hollow needle syringe is used as the cathode of the electrochemical workstation (ECW, CH Instruments Inc.) and the Au layer is electroplated in the Au sulfite solution (Yuncai Taotao Co., Ltd.). The hollow syringe is then washed using ethanol and dried in an oven (80 degrees C). Inner wall of hollow needles plated are then insulated using poly-methyl methacrylate (PMMA). Using a syringe, an ethyl acetate solution containing PMMA are slowly injected into the hollow needles, and the hollow needles are then dried in an oven (80 degrees C). To prepare the substrate of the microneedle array, the 3d-printed resin plate is cut by YLP-F Series Optical Fiber Laser marking Machine (Han's Laser Technology Industry Group Co., Ltd.). Then, holes with a 400 μm diameter are drilled in the 3d-printed resin plate to form the substrate with holes. The substrate is then washed in ethanol and dried in an oven (60 degrees C).

***Pre-position of microneedle arrays:*** According to the designed patterns, custom polyimide (PI) electrodes (Gaoyue Co., Ltd.) are laminated and patterned to the support structure with holes for microneedle integration. Silver wires with 0.5 mm thick are attached to the wielding pads of the PI electrodes. The hollow needles are inserted into the PI electrode and the support structure sequentially, with the orientation and needle length adjusted, and the position fixed. Then, silver wire soldered to the pads was then wrapped around the outer walls of the hollow microneedles and glued in place with silver glue (Yuncai Taotao Co., Ltd.). The hollow microneedles inserted on the substrate are not yet adhered and fixed to the substrate since the soldered silver wire retained sufficient length in advance, resulting the adjustable length of the hollow microneedle exposed under the substrate. With the Au electrode as the anode, the hollow microneedle electrodes are connected to the cathode of the electrochemical workstation and plated in an Au sulfite solution for 1200 s for the plating of Au layer of the outer wall.

***Preparation of working electrode:*** To construct the adhesion layer on the outer wall of the hollow microneedle electrode, Nafion solution containing Single Wall Carbon Nanotube (SWCNT) are prepared by mixing the SWCNT solution with the Nafion117 (Sigma) at a volume ratio of 1: 4. With the platinum sheet electrode as the anode, the hollow microneedle electrodes are connected to the cathode of the electrochemical workstation and plated in the Nafion solution containing SWCNT at 0.6 V for 60 s, before washing and overnight drying in room temperature. To prepare the hollow microneedle glucose sensing electrode, PBS phosphate buffered salt solution (pH = 7) containing GOx (50 mg/mL), osmium mediator solution (~10 mg/mL, Bioecare Technologies, Hangzhou Co. Ltd) and glutaric dialdehyde (2.5 mg/mL, Bioecare Technologies, Hangzhou Co. Ltd) are mixed at a volume ration of 4:14: 3 and shaken uniformly to obtain 2 mL of enzyme solution. The hollow microneedle electrode modified by Nafion is dip coated with the enzyme solution, uniformly lifted, and then air-dried for 16 h at room temperature. Polyurethane (PU, Aladdin) is added into a mixture of Tetrahydrofuran and Dimethylformamide (Tetrahydrofuran, THF, Dimethylformamide, DMF, both from Sigma) at a mass ratio of 98: 2. Uniformly shaken and overnight sonicated are required until the polyurethane solution is obtained for the dip-coating of the hollow microneedle electrodes. Additionally, to maintain the activity of the sensing layer, the prepared hollow microneedle glucose sensing electrodes should be stored at 4 ^o^C when not in use.

***Preparation of microneedle counter electrode and reference electrode:*** The hollow microneedle counter electrodes are prepared by connecting the hollow microneedle electrode to the cathode of the electrochemical workstation with the platinum sheet electrode as the anode for electrodeposition. The microneedle electrodes are electroplated in platinum sulfite solution (Yuncai Taotao Co., Ltd.) for 1200 s until a platinum layer is deposited on the outer wall of the electrodes.

For the preparation of hollow microneedle reference electrodes, polyvinyl butyral (PVB, Sigma) and sodium chloride (NaCl, Sigma) are added into methanol (Sigma) before shaking and sonicated overnight at 40 degrees C to obtain methanol solution containing PVB. The Ag/AgCl ink (Yuncai Taotao Co., Ltd.) are uniformly coated on the outer wall of the Au-plated hollow microneedle electrode, before drying in an oven (90 degrees C) for 1 hour. Methanol solution containing PVB are utilized for the dip-coating of electrode coated with Ag/AgCl ink before air-drying at room temperature for 16 hours.

***Integration of IEFMN:*** After modification of the hollow microneedle working electrode, counter electrode, and reference electrode, the orientation, position, and needle length of each electrode are adjusted so that the length of the exposed microneedles under the support structure are all 800 μm. Silver glue (Yuncai Taotao) are utilized to coat and adhere the silver wire to connect the electrode to the PI electrode. Then, the surface of the PI electrode is coated with 2% PMMA solution to achieve the purpose of insulating the solder pads and the back of the electrode.

***Morphological characterization of the hollow microneedle electrode:*** An optical microscope (Minmax) is used to optically characterize the prepared hollow microneedle electrode before and after modification. The surface morphology of the prepared hollow microneedle electrode is further characterized using SEM Pro (PHENOMSCIENTIFIC), with or without modification. Due to the difference in conductivity of the modified multilayer film structure on the surface of the hollow microneedle electrode, a layer of Au atoms (~10 nm thick) is deposited on the electrode surface for easier observation.

***Electrochemical performance test of hollow microneedle electrodes:*** the electrochemical performance of hollow microneedle electrodes is characterized in an *in vitro* environment by adding PBS phosphate buffered salt solution to a beaker covered with a four-folded sealing film to simulate skin. Hollow microneedle electrodes are utilized to pierce the sealing film to simulate the transdermal process. The hollow microneedle electrode is connected to CHI 760E electrochemical workstation and a series of electrochemical characterizations are performed to evaluate the performance of the working electrode with a commercial Ag/AgCl electrode as the reference electrode and a commercial platinum electrode as the counter electrode. The electrode is immersed in PBS buffer solution and CHI 760E are employed at a scan rate of 0.1 V/s to record the cyclic-voltage curve of the hollow microneedle electrode in PBS buffer solution to determine the working voltage in the I-t test. After the cyclic-voltage test, I-t test is performed on the hollow microneedle glucose electrode to record the time-current curve, with keeping the device unchanged. Glucose is gradually added to the PBS buffer solution, allowing the glucose concentration in the solution to vary as 3 mM/6 mM/9 mM/12 mM/15 mM/18 mM/21 mM/24 mM, with the I-t test repeated to record the time-current curves over a period.

***Optimization of electrochemical performance for hollow microneedle electrodes:*** Furthermore, the electrochemical performance of the electrodes is optimized by adjusting the ration of CNT doping in the sensing layer. Circular voltammetry curves are separately obtained for the MN/Au electrode, MN/Au/CNT/GOx electrode, MN/Au/20% (v/v) CNT/Wired-GOx/PU electrode, MN/Au/40% (v/v) CNT/Wired-GOx/PU electrode and the MN/Au/Wired-GOx/PU electrode. The amperometric response to different glucose concentrations is recorded. Additionally, to validate the electrochemical detection performance of multiple parallel-connected hollow microneedle glucose sensing electrodes, cyclic voltammetry curves of single needle, dual needles, and triple tandem arrangement of hollow microneedles are recorded in PBS phosphate buffer solution via electrochemical workstation, respectively.

***Specific response of IEFMN to glucose:*** After the cyclic-voltage test, I-t test is performed to record the time-current curve of IEFMN to PBS buffer solution containing glucose vary from 3 mM to 24 mM. Selective testing is conducted via electrochemical workstation. A series of selective tests are performed in PBS phosphate buffer solution by adding 10 mmol/L ascorbic acid, 0.2 mmol/L cholesterol, 2 mmol/L uric acid, 0.1 mmol/L sodium chloride (representing the salt solution), 0.1 mmol/L lactic acid, and 5 mmol/L glucose sequentially. The current response magnitude within 300 s is recorded. Additionally, a normalization analysis is carried out by setting the current change value after adding the glucose solution as 100%, thereby normalizing the current changes caused by various interferents.

***Electrochemical performance characterization of IEFMN:*** the electrochemical performance of hollow microneedle electrodes with PCBs attached to printed circuit boards is characterized in an *in vitro* environment. PBS phosphate buffered salt solution is added to a beaker and then covered it with a four times folded sealing film to simulate skin. The sealing film is pierced with a hollow microneedle electrode to simulate the skin penetration process. The IEFMN is connect to a printed circuit board and perform a series of electrochemical characterizations to evaluate the electrochemical properties of the IEFMN. Glucose is added gradually into the PBS phosphate buffered salt solution and allow the glucose concentration in the solution vary from 3 mM to 24 mM, with the current changes being recorded over a period of time.

***Characterization of the penetration performance of the hollow microneedle electrode:*** 2 mg/mL solution of rhodamine B is prepared to verify the penetration performance of the hollow microneedle electrode. Cotton swabs are used to absorb a small amount of the solution and the tip surface of the hollow microneedle array are uniformly colored. Human skin is replaced with fresh porcine skin, and the tip of the hollow microneedle array is then vertically placed on the surface of the porcine skin before firmly being inserted the microneedle array into the porcine skin for several minutes until pulling out. Subsequently, the pig skin is observed using an optical microscope. In addition, to verify the skin penetration performance of the hollow microneedle electrode, a razor blade is used to dissect along the cross-section of the hollow microneedle electrode puncture site to obtain a thin slice of the penetrated pig skin (approximately 200 μm). Then, the skin slice is placed under a fluorescence microscope and observed it with red fluorescence.

***Design and preparation of micro-channel module:*** To prepare suitable micro-channel module, an aluminum positive mold is designed (Ouyang Company) and polydimethylsiloxane (PDMS) is used to invert the positive mold of the workpiece to obtain the PDMS negative mold and a thin barrier layer. Then, the negative mold and the barrier layer are bonded using PDMS to form the microfluidic cavity. Next, the hollow microneedle electrode tubing opening is integrated with the microfluidic cavity and further connected to the infusion pump, microfluidic cavity and reservoir with the infusion tubing, respectively.

***Drug delivery of microneedle devices:*** the actual density of deionized water is weighed before testing the drug delivery performance of IEFMN. The printed circuit board is connected to the peristaltic pump, reservoir, IEFMN and power supply sequentially, and then the duty cycle of the excitation signal is adjusted in the circuit system to output the excitation voltage with a gradient. The peristaltic pump delivered liquid to the deionized water driven by the gradient excitation voltage (2.5 V/3 V/3.5 V/4 V/4.5 V/5 V). The amount of liquid delivered by the peristaltic pump in 1 minute are collected and analyzed statistically.

***Simulation of drug delivery of microneedle devices:*** In order to validate the drug delivery effect of IEFMN, the drug delivery of IEFMN is simulated by multi-physics field model of COMSOL software 6.0. The Transport of Diluted Species and Laminar Flow module of COMSOL software are employed to simulate the drug delivery process of the IEFMN. Initially, a 3D simulation model is established to represent the physical scenario of the hollow microneedle array penetrating the skin layer for drug delivery. Based on the geometric shape and dimensions of the 3x3 arrayed hollow microneedle devices and the skin tissue, separate 3D models are created for the hollow microneedles, the skin layer, and the subcutaneous tissue layer. Each individual hollow microneedle processed a length of approximately 0.8 mm, an outer diameter of 0.45 mm, an inner diameter of 0.37 mm, and a beveled tip angle of 30°. The top entry and bottom exit of each microneedle remained open to allow drug flow within the microneedle channels. The nine hollow microneedles are arranged with a spacing of 3 mm. The thickness of the stratum corneum in the skin layer is set at 0.2 mm, while the subcutaneous tissue layer had a thickness of 8.8 mm. The drug molecules are allowed to diffuse uniformly within the subcutaneous tissue, with an open boundary at the subcutaneous layer to permit free movement of drugs beyond the boundary. The section of the hollow microneedle array that penetrated the skin layer and entered the skin tissue is approximately 0.8 mm in length. To simulate drug delivery, the top entry of the hollow microneedle array is connected to the drug delivery system. At this entry point, a high-molecular-weight solution (mimicking insulin solution) with a molecular weight of 6000 is introduced, with an initial concentration of C₀c = 1 mol/m³. By adjusting the drug injection flow rate, the rate at which drugs are delivered through the hollow microneedle array into the subcutaneous tissue is controlled. This simulation aimed to replicate the scenario where insulin, under the influence of a peristaltic pump, is transported through the hollow microneedle channels of the array into the subcutaneous tissue. Also, to verify the drug delivery efficiency of IEFMN, the delivery efficiency is evaluated by calculating the insulin concentration in the whole subcutaneous tissue under different time conditions and summing the integrals. The concentration of the delivered insulin solution is normalized by normalizing the initial insulin concentration in the interstitial fluid to the delivered insulin solution.

The relevant physical parameters used for the simulation are shown in Chart I:

Chemical Species Transport module of the software is utilized by invoking the dilute species transport interface to simulate the dynamic transport process of chemical components. The material adheres to the conservation of mass equation (1):


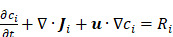

(1)

Equation (1) encompasses the mechanisms of diffusion and convection transport, where c_i_ represents the concentration of the substance, ▽ denotes the Hamiltonian operator, R_i_ is the expression for the reaction rate of the substance, and u is the average velocity vector of the material. Since molecular diffusion often triggers mass transfer at the dilute species transport interface, the diffusion flux vector is defined by the mass flux J_i_. J_i_ is directly correlated with the conservation of mass equation (1) and can be used to calculate flux and boundary conditions, where D_i_ stands for the diffusion coefficient.


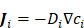

(2)

By invoking the laminar flow interface within the simulation software's fluid flow module, the computation of velocity fields and pressure fields for drug flow under laminar conditions can be conducted. The governing equations of the laminar flow interface are primarily based on the Navier-Stokes equation, with its general form being:


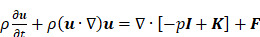

(3)

Where equation (3) is a vector equation that represents the conservation of momentum. Equation (4) represents the continuity equation, signifying mass conservation. Equation (5) pertains to the solution of viscous stress tensor, facilitating the computation in conjunction with equation (3). Here, ρ signifies density, u stands for velocity vector, p represents pressure magnitude, k denotes viscous stress tensor, and F denotes the volume force vector.


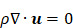

(4)


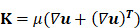

(5)

***Drug delivery via IEFMN:*** *in vivo* experiments are performed with rats to evaluate the role of IEFMN in blood glucose monitoring in animals. This study is approved by the Animal Management and Use Committee of Sun Yat-sen University. All animals are cared for humanely according to institutional guidelines. SD-IGS rats are obtained from the animal laboratory of Sun Yat-sen University and used them for the experimental study. The animals used included 2 diabetic rats. First, the rats are anesthetized with gas, and then surgical scissors and depilatory cream are used to depilate the back of the rats to obtain bare skin with an area of approximately 3 × 4 cm^2^. Subsequently, the IEFMN is placed on the depilated area so that the IEFMN is in close contact with the skin and penetrated the stratum corneum. At the beginning of the experiment, 1.2 mL of insulin solution (8 IU) is delivered to the rats via IEFMN over 60 min. And, blood is taken from the tail artery of the rats every 15 minutes and the glucose level in the blood of the rats is measured with a glucometer. The experiment is conducted for a total of 2 days, lasting 3 hours each day. After the experiment is completed, the rats are put back in their cages.

***In vivo sensing experiments with microneedle devices:*** *in vivo* experiments are conducted with rats to evaluate the role of the IEFMN system in animal glucose monitoring. This study is approved by the Animal Management and Use Committee of Sun Yat-sen University. All animals are cared for humanely according to institutional guidelines. SD-IGS rats are obtained from the animal laboratory of Sun Yat-sen University and used them for the experimental study. The animals used included three healthy rats and three diabetic rats. First, the rats are anesthetized with gas and then surgical scissors and depilatory cream are used to depilate the back of the rats to obtain bare skin with an area of approximately 3 × 4 cm^2^. Subsequently, the IEFMN is placed over the depilated area so that the IEFMN is in close contact with the skin and penetrated the stratum corneum. The IEFMN is connected to an electrochemical workstation and recorded the current measured by the IEFMN for 300 s at an operating voltage of 0.15 V. Recordings are made every 15 min for 3 h. In this study, we performed parallel experiments on 2 groups (healthy group and diabetic group, 3 rats each), for a total of 6 rats, each rat repeated for 3 days. For healthy rats, 3 mL of glucose solution (5% by weight) are injected subcutaneously by syringe at the 90th minute; for diabetic rats, 1.2 mL of insulin (8 IU) solution are injected. The measured current values are converted to glucose concentrations according to the current-metabolite concentration standard curve of IEFMN. Every 15 minutes, blood from the tail artery of the rats is collected, and then the glucose level in the blood of the rats is measured with a glucometer. After the experiment is completed, the rats are put back in their cages.

***Design of printed circuit boards:*** the circuit system is designed and integrated, with circuit printing performed and component soldering to obtain the printed circuit board. The prepared circuit board is connected to the IEFMN through the sensor interface and to the peristaltic pump through the row of wires, before being placed in the printed box and then fixed. When in use, the battery module drove the board to collect the current signal from the IEFMN and process the signal through a two-stage differential circuit to reduce signal distortion and common mode noise interference. Subsequently, the STM32 chip converted the collected analog signal into a digital signal via an ADC and performs gradient conversion to obtain the corresponding glucose concentration based on the calibration curve obtained from *in vitro* experiments. The processed glucose concentration changes are transmitted to the Bluetooth module via the data serial port and then sent to the mobile terminal. A graph reflecting the changes in glucose levels is displayed in real time on an interface designed by the LabVIEW software.

## Supplementary Material

Supplementary figures.

## Figures and Tables

**Chart I Relevant:** physical parameters utilized in simulation.

Symbol		Definition
ρ_b_	318.18 kg/m^3^	Density of bovine insulin
μ_b_	0.0001 Pa · s	Dynamic viscosity of bovine insulin
ρ_f_	1300 kg/m^3^	Density of dermal interstitial fluid
μ_f_	2 Pa · s	Dynamic viscosity of dermal interstitial fluid
D_d_	25 x 10 ^-11^ m^2^/s	Diffusion coefficient of dermis
D_e_	25 x 10 ^-11^ m^2^/s	Diffusion coefficient of epidermis
D_i_	5 x 10 ^-11^ m^2^/s	Diffusion coefficient of insulin
T	293.15 K	Temperature
P	0	Pressure
