# Supplementary material for: Integrated electronic/fluidic microneedle system for glucose sensing and insulin delivery
Source: Theranostics. 2024 Feb 11;14(4):1662–82. doi: 10.7150/thno.92910 (PMC10879877; doi:10.7150/thno.92910)
Supplement: Supplementary file 1 — Supplementary figures. [file thnov14p1662s1.pdf]

Integrated electronic/fluidic microneedle system for glucose sensing and insulin delivery

Supplementary Material

Xinshuo Huang, Baoming Liang, Shuang Huang, Zhengjie Liu, Chuanjie Yao, Jingbo Yang, Shantao Zheng, Feifei Wu, Wan Yue, Ji Wang, Huijiuan Chen, * Xi Xie *

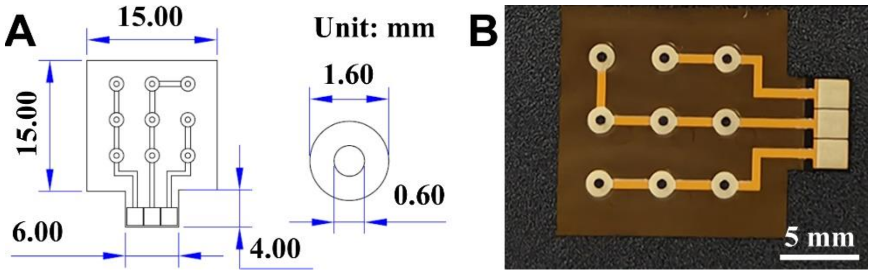

Figure S1. (A) Design and (B) photographic of the PI thin-film circuit for IEFMN connection. The designed PI thin-film electrode has dimensions of 15 mm in length, 15 mm in width, and a thickness of 150 μm. The electrode features three channels with a total of nine pads arranged in a 3x3 array. Each pad in the channels has an outer diameter of 1.6 mm, an inner diameter of 0.6 mm, and a spacing of 3 mm between the center points. The working electrode channels can be connected in series with three hollow microneedle glucose sensing electrodes, the counter electrode channels can be connected in series with four hollow microneedle counter electrodes, and the reference electrode channels can be connected in series with two hollow microneedle reference electrodes. After integration with the hollow microneedles, the PI thin-film electrode is connected to the interface on the PCB through gold fingers to achieve stable signal transmission.

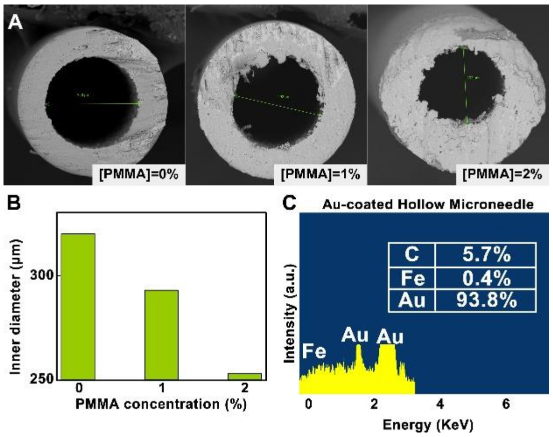

Figure S2. (A) Comparison of internal diameters of hollow microneedles modified by different concentrations of PMMA. (B) For hollow microneedles without PMMA modification, the inner diameter is about 320 μm. For the hollow microneedles modified with 1% PMMA, the inner diameter is about 293 μm. For the 2% PMMA coated hollow microneedles, the inner diameter is about 253 μm. **(C) EDX spectrum of the Au-coated hollow microneedle electrode.** The EDX spectrum suggests that there is no presence of osmium (Os) on the surface of the hollow microneedle electrode before modification.

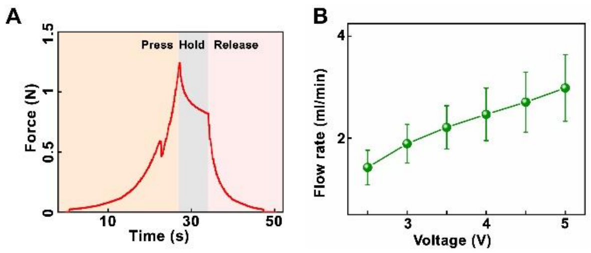

Figure S3. (A) Stress-strain curve observed during the penetration of the IEFMN into the skin. The result suggest that the force gradually increased during the insertion of the hollow microneedle into the skin, with a puncture force of approximately 0.53 N. **(B) Delivery amount within 10 mins using IEFMN with different flow rates of IEFMN in different excitation voltage**. The volume of solution delivered by the IEFMN increased linearly with time, and the increase in driving voltage also significantly increased the volume of solution delivered by the IEFMN. As the excitation voltage was gradually increased from 2.5 V to 5 V, the flow rate of IEFMN was gradually increased from 1.42 ml/min to 2.98 ml/min.

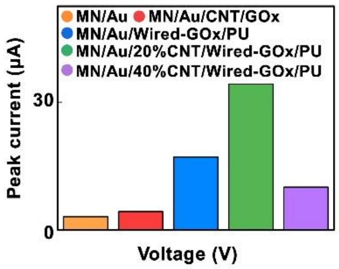

Figure S4. Peak current statistics of the working electrode with varying component ratio doping in the sensing layer. The peak currents for MN/Au electrode and MN/Au/Wired-GOx/PU electrode is 2.00 μA and 2.81μA, respectively. For the MN/Au/Wired-GOx/PU electrode, current maximum appears to be 11.36 μA. As for the MN/Au/20%CNT/Wired-GOx/PU electrode and the

MN/Au/40% CNT/Wired-GOx/PU electrode, current maximum appears to be 22.83 μA and 6.56 μA, respectively.

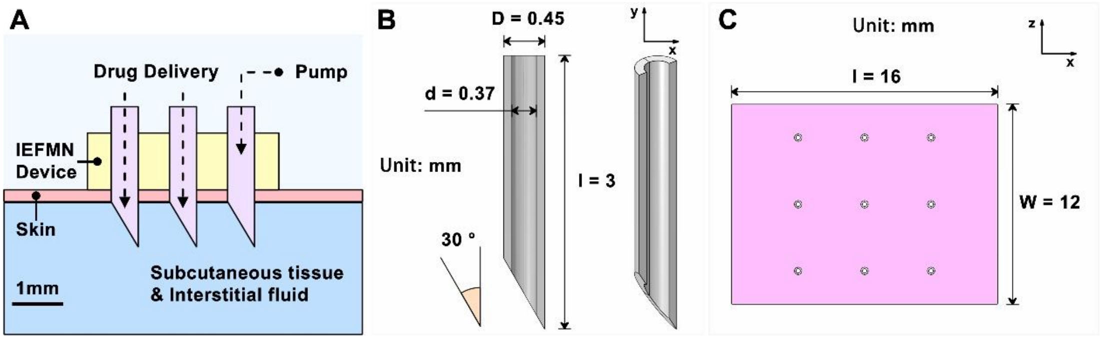

Figure S5. Schematic of the IEFMN for subcutaneous drug delivery. (A) Diagram showing the penetration of hollow microneedle electrode array through the skin layer and drug delivery through the inner wall channel. (B) Cross-sectional view and detailed parameters of a single hollow microneedle constructed in the software, including length of 0.8 mm, outer diameter of 0.45 mm, inner diameter of 0.37 mm, and a bevel angle of 30° in the cross-sectional needle head. (C) Top view and detailed parameters of the simulated skin layer model in the software. The dimensions of the skin layer are 16 mm (length) × 12 mm (width) × 0.2 mm (thickness).

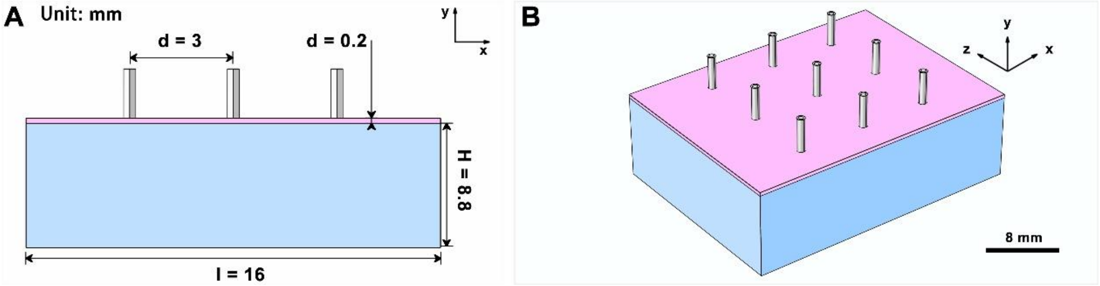

Figure S6. Schematic of the IEFMN for subcutaneous drug delivery. (A) Side view and detailed parameters of the simulated three-dimensional drug delivery model. The dimensions of the subcutaneous tissue layer are 16 mm (length) × 12 mm (width) × 8.8 mm (thickness), and the drug delivery molecules diffuse uniformly within the subcutaneous tissue. (B) Overview of the three-dimensional simulated model of IEFMN for molecule delivery.

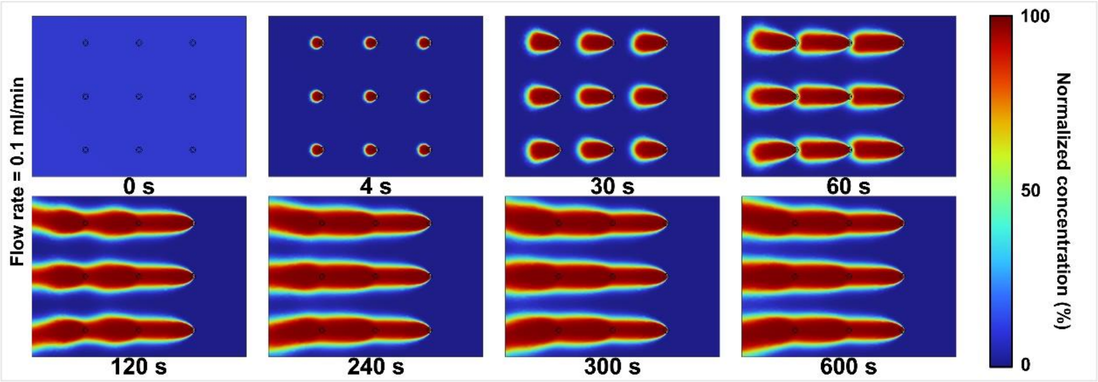

Figure S7. Schematic of the IEFMN for subcutaneous drug delivery. Concentration distribution of molecules in the xz direction of skin tissue during drug delivery at a flow rate of 0.1 mL/min for the initial 600 seconds.

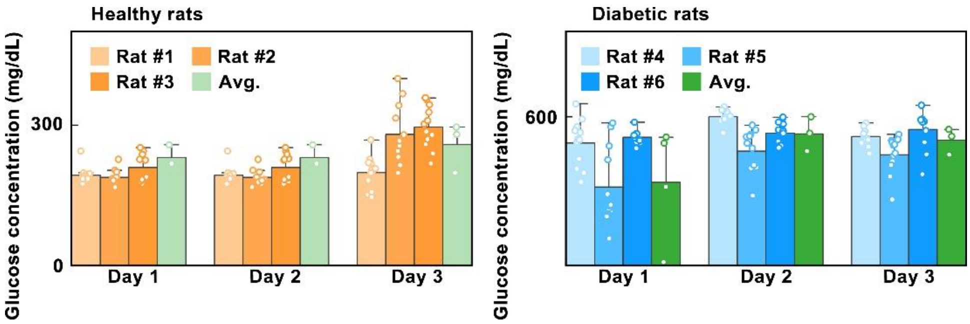

Figure S8. Graph showing the average daily blood glucose level for each rat via 3-point calibration over 3 days. N = 6 samples. Over a three-day period, diabetic rats had significantly greater blood glucose fluctuations than healthy rats.

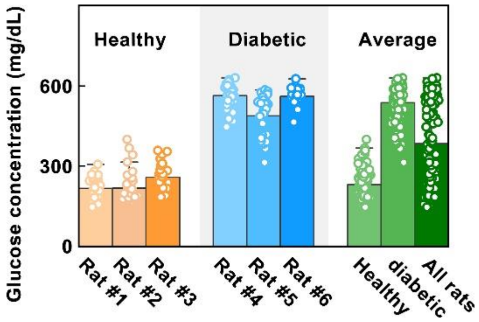

Figure S9. Graphs showing the average blood glucose level of each rat via 3-point calibration over 3 days. n = 6 samples. The average blood glucose concentrations of three healthy rats are 216.5 mg/dL, 217.5 mg/dL, and 257.8 mg/dL, respectively. For diabetic rats, the average blood glucose concentrations are 563.7 mg/dL, 487.8 mg/dL, and 560.8 mg/dL.

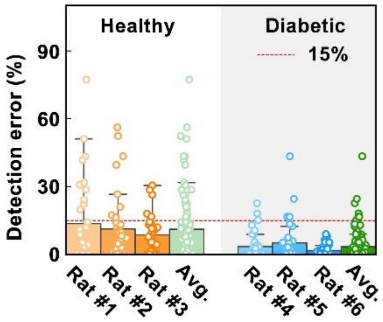

Figure S10. Statistical analysis showing the detection error of the IEFMN for different rats with 3-point calibration during 3 days. N = 6 samples. Most of the data points are located in the region where the error is less than 15%. The detection error for diabetic rats is slightly less than that for healthy rats, this may be attributed by higher blood sugar levels in the diabetic rats themselves.

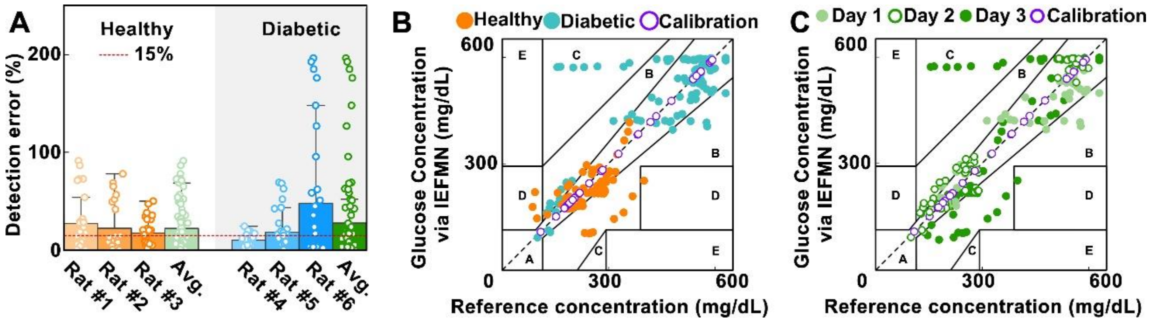

Figure S11. Error analysis of data measured using IEFMN through 2-point calibration. (A) Statistical analysis showing the detection error of the IEFMN with 2-point calibration for different rats during 3 days. N = 6 samples. In both healthy rats and diabetic rats, detection error of the IEFMN exceed 15%, which is unacceptable clinically. Clarke's error grid analysis showing the detection accuracy of glucose by IEFMN through 2-points calibration, compared to the actual BGLs measured via standard glucometer. The analysis is divided by experimental group (B) and experimental time (C). The error range for each region is: region A <20%, B 20-50%, C 50-80%, D 80-100%. Whether by experimental group or experimental time, data appeared in regions C and D, suggesting potential clinical risk in clinical usage. Data outside the normal range (less than 0 mg/dL or more than 650 mg/dL) are not included.

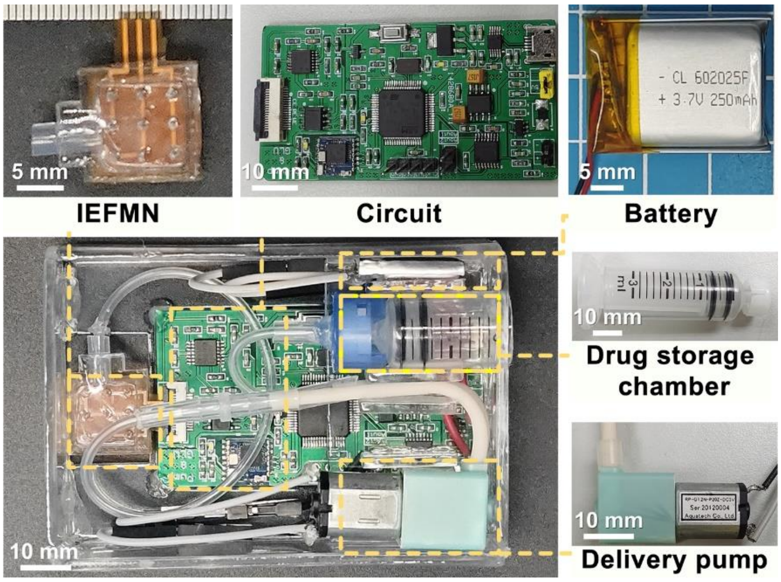

Figure S12. Graph showing the component details of the IEFMN-based system, including the IEFMN, battery, supporting circuit, delivery pump and drug storage chamber. The IEFMN patch is connected to the printed circuit board via flexible PI electrode. Delivery pump and the drug reservoir are connected via a hose. A battery on the circuit board drives the microprocessor to motivation to the circuit, achieving signal collection and on-demand drug release via the delivery pump.

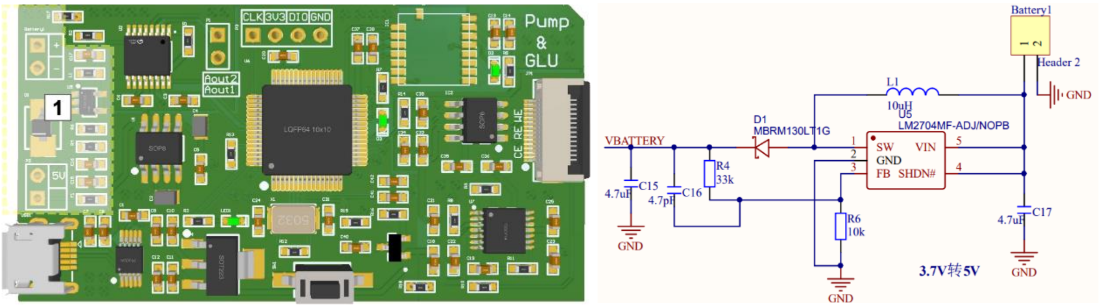

Figure S13. Lithium-ion Battery-Powered Module. Employing a low-power step-up DC/DC converter chip, this module elevates the stable 3.7V voltage from the lithium-ion battery to 5V, providing the operational voltage for the signal conditioning module's operational amplifier.

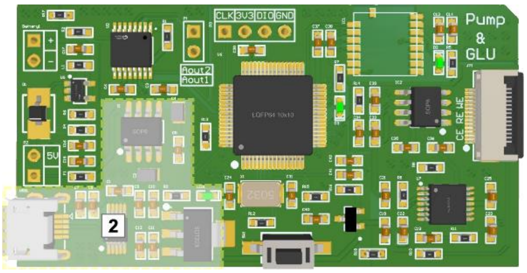

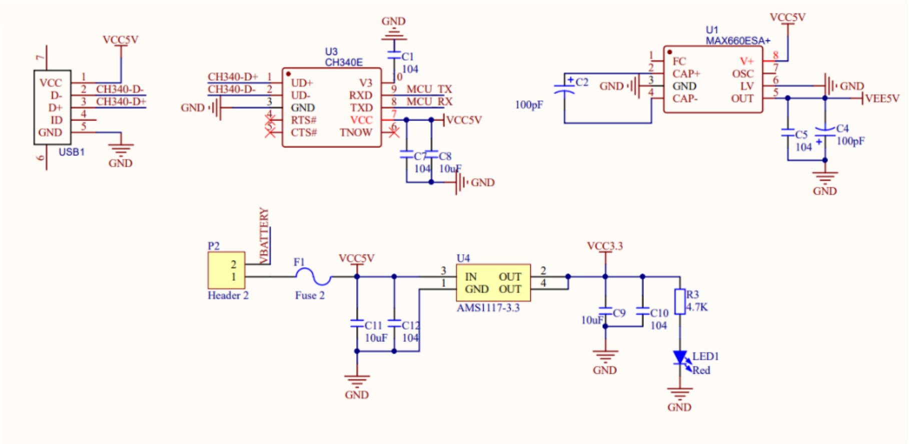

Figure S14. USB Serial Interface and Power Conversion Module. This module comprises two key components - a USB serial interface module and a power conversion module. The USB serial interface module employs the CH340E USB-to-Serial chip, enabling the upgrading of the MCU's serial communication to the USB bus. Additionally, serving as a power supply module, it provides a 5V voltage source to power various modules on the circuit board, facilitating their debugging. The power conversion module includes two sections: a 5V to -5V conversion (utilizing MAX660ESA) and a 5V to 3.3V conversion (using AMS1117-3.3). The -5V voltage is employed to supply the necessary voltage for post-stage operational amplifiers and motor driver chips to operate effectively. Meanwhile, the 3.3V voltage serves as the required power source for the MCU's normal operation.

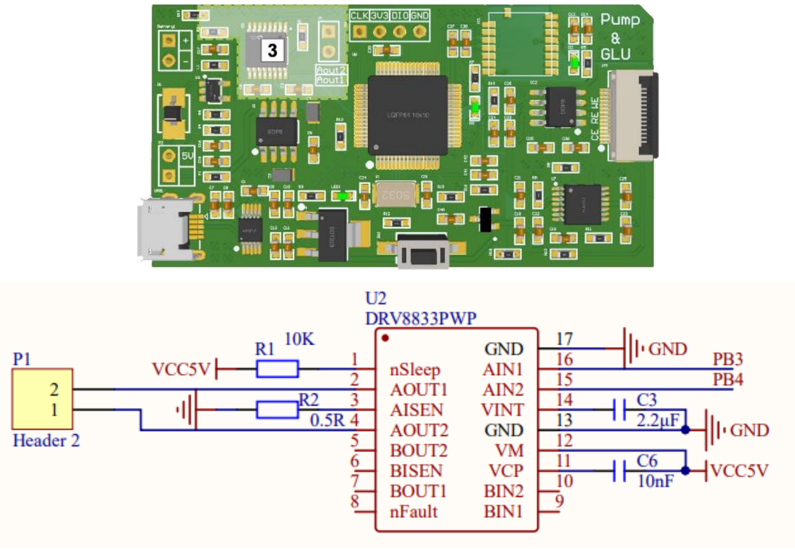

Figure S15. Motor Drive Module. This module employs the DRV8833PWP motor driver chip to control motor speed through PWM waveforms with varying duty cycles provided by the MCU.

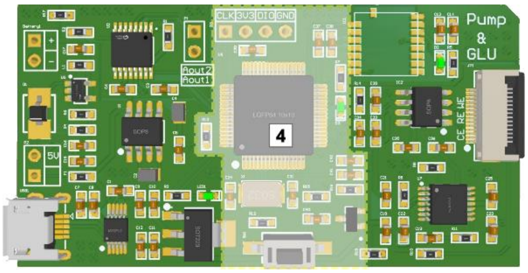

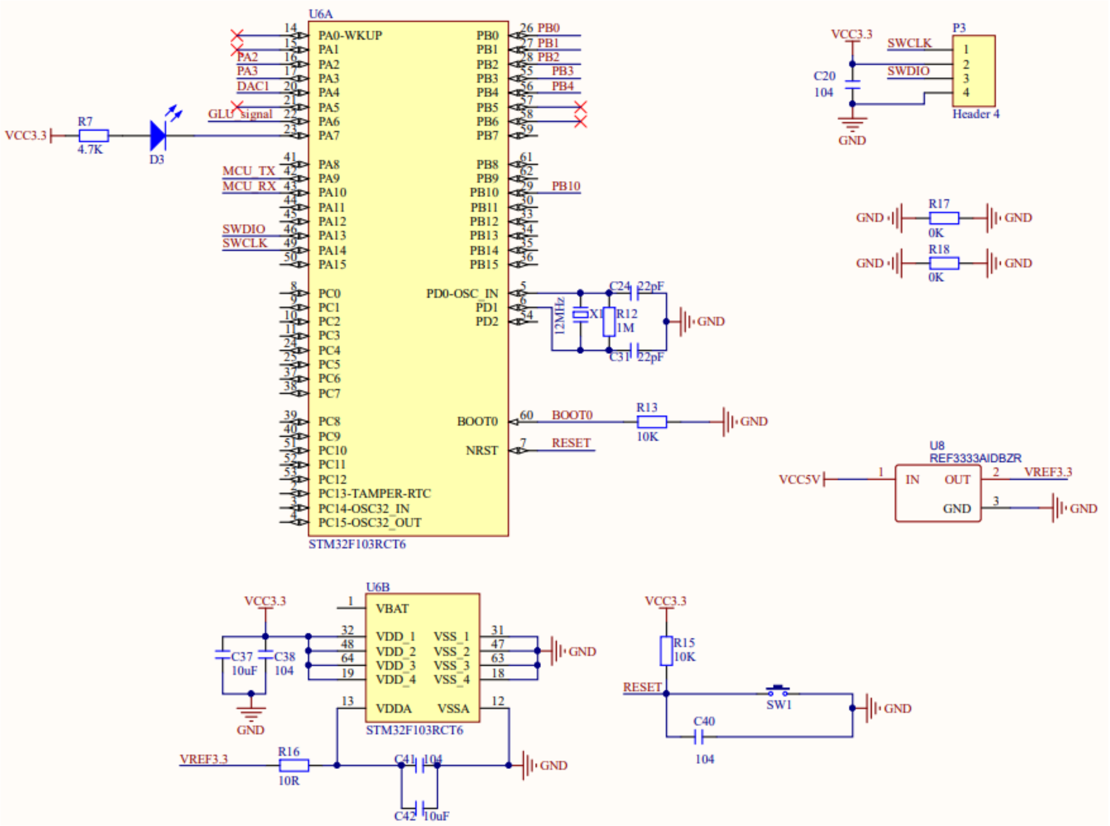

Figure S16. Minimal System Module of the Microcontroller. This module efficiently leverages resources within the main control chip (STM32F103RCT6) to facilitate data transmission, system reset, and other essential functions. It encompasses the following components: 1) Crystal Oscillator Circuit: With an external 12MHz crystal oscillator, this component provides the precise clock signal required for system operation. 2) Download Circuit: Facilitating program downloads to the STM32 chip via the ST-link interface, enabling rapid program updates and modifications. 3) Reset Circuit: Achieves program reset functionality through a reset button press. 4) Data Transmission: This segment encompasses both inter-board data transmission and data transfer with a host computer. Inter-board data transmission involves the use of the DAC and ADC modules within the chip for voltage signal generation required by downstream test circuits. Additionally, it converts signals collected by the downstream circuitry into digital signals interpretable by the host computer. Data transmission with the host computer occurs via USB serial communication or Bluetooth module, each implemented through appropriate pin configurations on the chip.

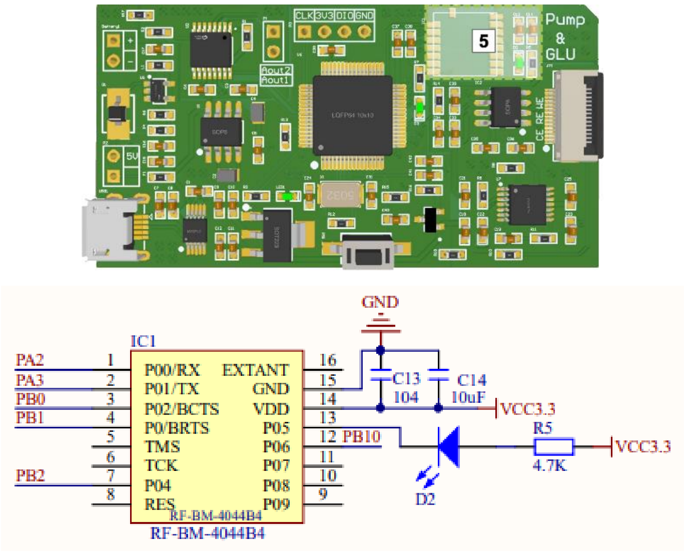

Figure S17. Integrated Low-Power Bluetooth Radio Frequency Module (RF-BM-4044B4). This module serves as the bridge connecting the STM32 to the host computer, enabling highly integrated wireless communication capabilities. It facilitates the convenient wireless transmission of data collected by the circuit to the host computer.

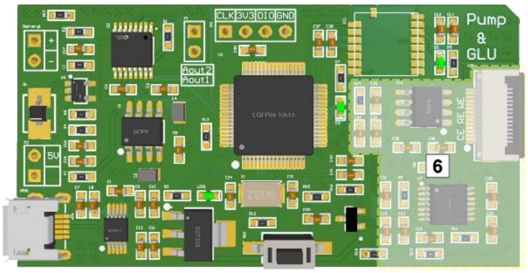

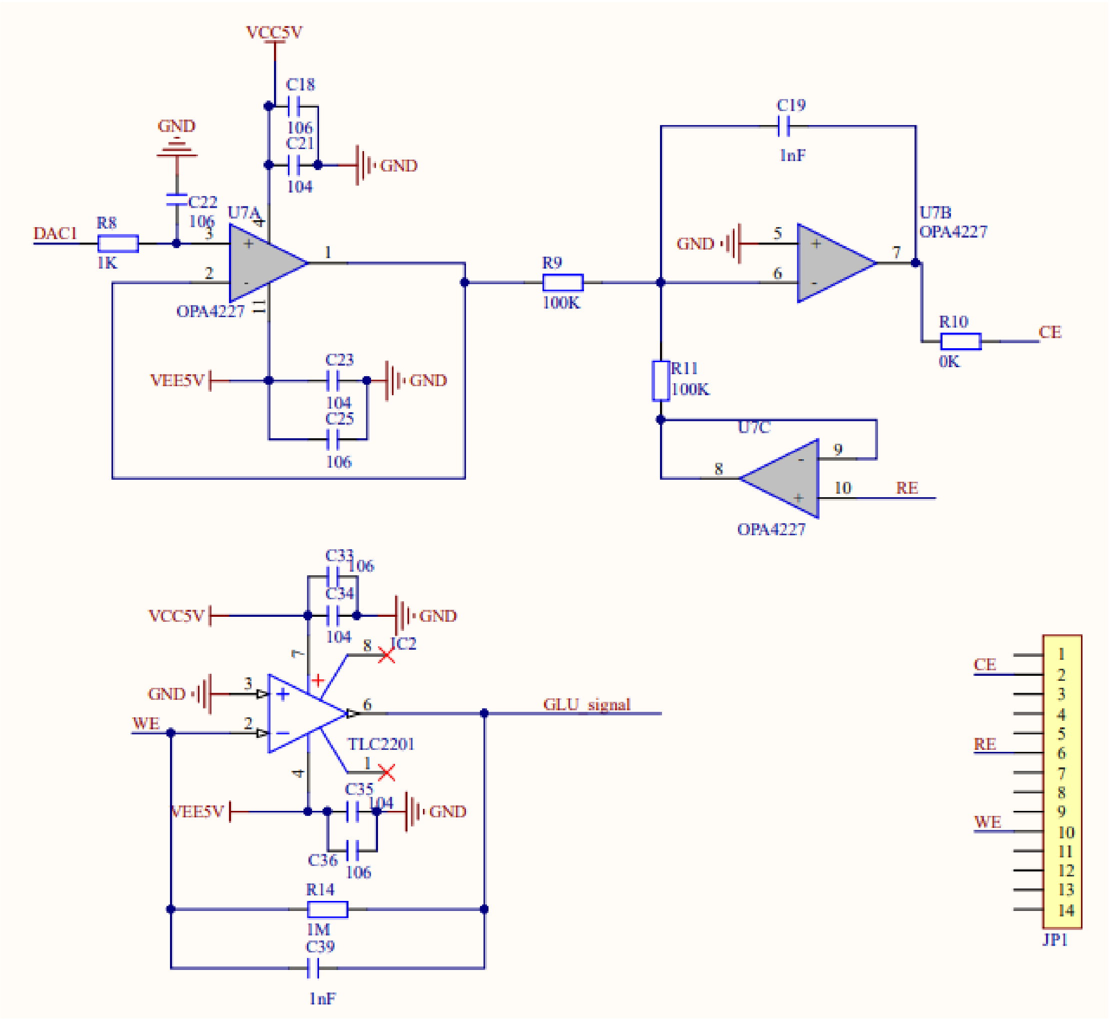

Figure S18. Detection Circuit. This module serves as the three-electrode sensing circuit for glucose data acquisition. It utilizes the MCU's DAC module to provide a 0.15V voltage input to the voltage follower. The first-stage voltage follower not only enhances the circuit's load capacity but also isolates and buffers the signal, further stabilizing the reference voltage and the operating voltage on the electrode. The working electrode employs a transimpedance amplifier circuit, employing the high-precision, low-noise operational amplifier chip (TLC2201) to convert the current value from the test solution into a voltage signal within the MCU's readable range.

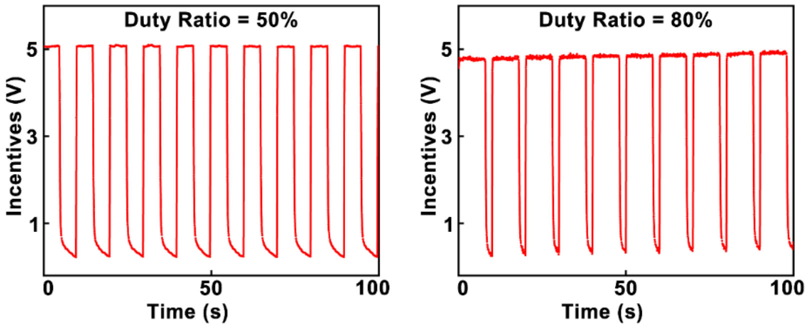

Figure S19. Images of PWM waves with different duty cycles (50% and 80%) generated by the MCU. By modulating the PWM output, the motor drive module controls the motor speed and consequently adjusts the flow rate of the peristaltic pump.
